# MicroRNAs modulate ethylene induced retrograde signal for rice endosperm starch biosynthesis by default expression of transcriptome

**DOI:** 10.1038/s41598-021-84663-2

**Published:** 2021-03-10

**Authors:** Sonam Panigrahi, Madhusmita Panigrahy, Ekamber Kariali, Sushanta Kumar Dash, Binod Bihari Sahu, Sushil Kumar Sahu, Pravat Kumar Mohapatra, Kishore Chandra Sekhar Panigrahi

**Affiliations:** 1 School of Life Sciences, Sambalpur University, Jyoti vihar, Sambalpur, 768019 India; 2 Siksha ‘O’ Anusandhan University, Bhubaneswar, 751030 India; 3 ICAR-National Rice Research Institute, Cuttack, 753006 India; 4 Department of Life Science, National Institute of Technology, Rourkela, 769008 India; 5 School of Life Sciences, Ravenshaw University, Cuttack, 753003 India; 6 School of Biological Sciences, National Institute of Science Education and Research, Khordha, 752050 India; 7 Homi Bhabha National Institute (HBNI), Anushakti Nagar, Mumbai, 400094 India

**Keywords:** Plant biotechnology, Molecular engineering in plants, Biotechnology, Plant sciences

## Abstract

Control of stage specific spike in ethylene production at anthesis has been a vauable route to potentially enhance genetic ceiling for grain filling of rice spikelet. A number of genes controlling ethylene homeostasis and starch synthesis have been identified so long, but lack of credible information on master modulation of gene expression by miRNAs and their target genes associated with hormonal dynamics obfuscate mechanisms controlling genotype difference in quantum of grain filling. The confusion accounts for consequent shrinkage of options for yield manipulation. In a two by two factorial design, miRNA regulation of spikelet specific grain development in low against high sterile recombinant inbred lines of rice *Oryza sativa* L. namely CR 3856-62-11-3-1-1-1-1-1-1 (SR 157) and CR 3856-63-1-1-1-1-1-1-1 (SR 159) respectively, and inferior verses superior spikelets were compared during first 10 days after anthesis. Grain filling was poorer in SR159 than SR157 and inferior spikelets in the former were most vulnerable. Between the cultivars, overall expression of unique miRNAs with targets on ethylene pathway genes was higher in SR159 than SR157 and the situation was opposite for auxin pathway genes. Precision analysis in psTarget server database identified up-regulation of MIR2877 and MIR530-5p having Os11t0141000-02 and Os07t0239400-01 (PP2A regulatory subunit-like protein and ethylene-responsive small GTP-binding proteins) and MIR396h having Os01t0643300-02 (an auxin efflux carrier protein) and Os01t0643300-01 (a PIN1-like auxin transport protein), as targets with highest probability at anthesis and 5 days after anthesis respectively, in the inferior spikelet and the fold change values of DGE matched with pattern of gene expression (relative transcript level) in the qRT-PCR studies conducted for relevant miRNAs and protein factors for ethylene and auxin signalling. In conclusion, epigenetic regulation of both auxin and ethylene homeostasis control grain filling of rice spikelet was established, but evidences were more robust for the latter.

## Introduction

For food calories, nearly half of the seven billion people on earth depend on rice^1^. Majority of the rice consumers live in the low income countries of tropical Asia and for them rice accounts for more than 76% of caloric intake^2^. Improvement of yield potential of the irrigated IR8-parented semi-dwarf rice has accounted for the food grain surplus in the rice growing nations and ensured food security since 1960s. However, the yield potential has almost stagnated in the last 2–3 decades and on farm yield seldom has gone beyond 10 t ha^−1^^3^ in spite of improved management practices. Currently speculation is ripe for a shortage of food in the absence of another quantum jump of yield potential in near future^4^. For gain in yield potential, breeders have targeted on improvement of panicle grain number. Using advanced technologies and accessing improved breeding populations, several new large panicle rice cultivars bearing numerous spikelets, such as, hybrid rice, super rice, new plant type rice have been developed^3,5,6^ but the intended matching benefit of grain yield is not accrued. The increase of spikelet/grain number without corresponding increase of size makes the panicle compact/dense, where a trade off between grain number and filling derides commensurate advancement of yield benefit^10^. Consequently the failing spikelets located especially on the proximal part of panicle become barren or poorly filled^7,8^. Spikelets compromised for accommodation within crammed space of panicle rachis produce high concentration of ethylene at anthesis to the detriment of starch synthesis and filling of the developing grains^9,10^.

Ever since ethylene being identified first time as the causal agent for poor grain development^11^, investigations have progressed into molecular biology of ethylene signal transduction for elucidation of transcriptome dynamics underplaying synthesis of the protein factors involved in the process^12^. It is now known that higher concentration of ethylene synthesized in the poorly developed inferior basal spikelets at early grain filling stage elicits over-expression of ethylene receptor and signal transducer proteins^13,18^ which underscores expression of genes encoding endosperm starch synthesizing enzymes and cell cycle regulators^10,14^. In contrast, superior spikelets located on the apical part of panicle are not subservient to ethylene action and produce high density well filled grains^15^. At anthesis neither they produce ethylene profusely nor there is over expression of genes encoding proteins for ethylene signal transduction^16^. It is surmised that spikelet specific ethylene production/action could be a factor sub-ordinate to IAA for the apical dominance in grain filling. Our earlier work has shown that IAA primarily promotes grain filling of apical spikelets of rice panicle^17^ and ethylene functions as a second messenger to transmit IAA signal slackening grain filling of basal spikelets^13^.

The variation in transcriptome dynamics among different panicle type cultivars, showing evidence for a co-ordinated action of ethylene and IAA is explicit^18^, but lacks consolidation conceptually to identify genetic factors involved in the spikelet specific grain filling/endosperm starch biosynthesis of rice panicle. It is possible that ethylene becomes a retrograde signal for endosperm starch biosynthesis^10^ by default expression of the genome for which epigenetic factors are responsible. Epigenetic factors like miRNAs control expression level of their target genes negatively^19^ through either pre-transcriptional modification by DNA-methylation or post-transcriptional alteration by cleavage or translational suppression of target mRNAs. Recently, there has been a plethora of studies conducted to isolate the epigenetic factors, especially the miRNAs expressing differently between the superior and inferior spikelets during grain development^20^. These differentially expressed miRNAs possibly participate in control of hormone metabolism, carbohydrate metabolic pathways and cell division of the developing kernels and difference in function and expression levels of the miRNAs determine the quantum of grain filling of the spikelets. To study the expression pattern during grain development, miRNAs from all growth stages were sequenced and novel miRNAs involved in the process have been identified^21^. Peng et al.^22^ analysed 457 known and 13 novel miRNAs and showed differential expression of 141 known miRNAs between superior verses inferior spikelets with higher level of expression associated in most of the cases in the former than in the latter. The genes targeted by these differentially expressed miRNAs were linked to signalling pathways of plant hormone homeostasis and starch synthesis during the storage phase of grain development; precisely day 10 after anthesis and onwards. However, information is scant on the precise role of the miRNAs in genome dynamics regulating target mRNA expression for hormone homeostasis during early part of caryopsis development, when ethylene-induced endosperm morbidity underscores grain development of failing spikelets of rice panicle in spite of advancement of test of small RNA sequencing repeatability of rice^23^. In our committed pursuit^9^ to unravel expression of genes encoding ethylene signal that infringe grain development of spikelets disadvantaged for their orientation in time and space on the panicle architecture of rice during the first 10 days after anthesis, the role of some of the miRNAs known or unknown so long have been investigated. Early part of grain development period provides license for fixing the cell number of endosperm and ultimate size of grain at maturity^16^. An experiment has been designed accordingly in a narrow genetic window to test the concept using recombinant inbred lines of rice contrasting for panicle grain number and fertility. To understand the differential pattern of regulatory network that co-ordinates gene expression programs and finalize developmental plasticity for grain filling between the contrasting spikelet types, dynamic variation of the miRNAs controlling expression of target genes specific to ethylene or auxin pathways have been selected for exploration.

## Materials and methods

### Generation of RILs, growth conditions and sampling

**Photo 1:**
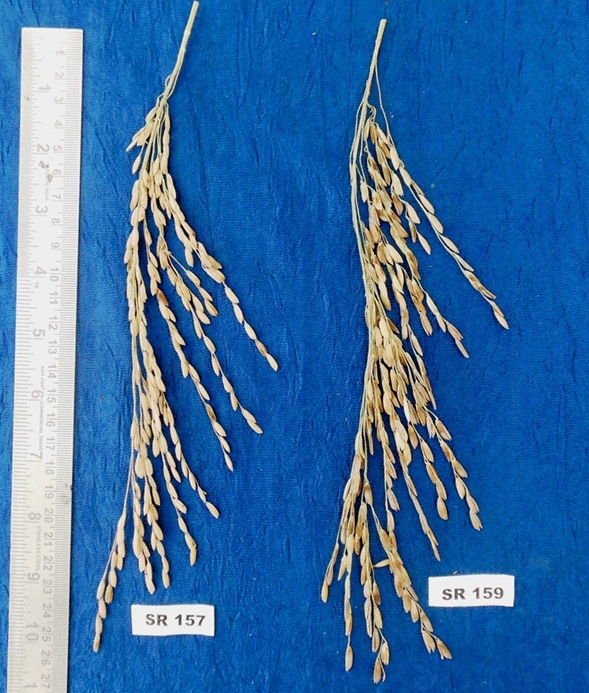
Photograph of panicle of recombinant inbred lines of rice: SR-157 (low sterile) and SR-159 (high sterile).

The recombinant inbred lines (RIL) of rice *Oryza sativa* L*.* used in this experiment were generated by cross breed of IR73963-86-1-5-2-2 and CR 2324-1 as parents and subsequent development of a mapping population. Of the two cultivars, the first one was selected from a lot of tropical japonica derived NPT line, with high general combining ability, whereas the second was a potential aromatic culture from ICAR-National Rice Research Institute (NRRI), Cuttack. Moderately large number of F2 population was generated from the cross and these lines were maintained using Single Seed Descent (SSD) up to F9 population. At this population level RIL genotypes were selected for the study viz., CR 3856-62-11-3-1-1-1-1-1–1 (SR 157) and CR 3856-63-1-1-1–1-1-1-1 (SR 159) (Photo 1) with contrasting grain filling patterns, i.e., high percentage of spikelet fertility in the former and low fertility in the latter. The RIL cultivars were grown in the irrigated field conditions of NRRI during the wet season of 2017 with recommended agronomic practices.

Spikelets on apical and basal part of panicle on main shoot of rice plant were excised and immediately frozen in liquid nitrogen at different stages of post-anthesis period. Spikelets on the upper-most two branches reaching anthesis first in the panicle were considered apical (superior) spikelets. The spikelets of lowermost two primary branches of panicle reaching anthesis 5 days later were basal (inferior) spikelets. The spikelets were collected on 0, 5 and 10 days after anthesis for both cultivars SR-159 and SR-157. The six samples, comprising three each of apical and basal spikelets in SR-159 were combined into Group-1. Similarly, the six samples of SR-157 were combined into Group-2.

### Measurement of morphological parameters

Morphological features like plant height, flag leaf area, panicle length and weight, number of primary and secondary branches, total length of primary and secondary branches, filled and unfilled grain number, grain density, primary and secondary branch density, sterility percentage of the panicle of the main shoot were measured at maturity stage in both the genotypes. Similarly tiller number per hill and 100 grain weight were also recorded at maturity (Table 1).

**Table 1:** Plant morphology and grain yield parameters of panicle on main shoot of two RIL rice lines SR-159 (sterile) and SR-157 (fertile) grown in the field conditions at NRRI, Cuttack during the wet season of 2017.

RIL rice lines	Plant height (cm)	Flag leaf area (cm^2^)	No. of tiller	Panicle length (cm)	Panicle weight (g)	100 well filled grain weight (g)	No. of primary branches	Total length of primary branches (cm)	No. of secondary branches	Total length of secondary branches (cm)	No. of filled grains	No of unfilled grains	Grain density	Primary branch density	Secondary branch density	**Sterility (%)**
SR-159	100.00 ± 0.81	43.23 ± 0.85	8.33 ± 0.47	26.33 ± 0.704	1.716 ± 0.77	2.428 ± 0.09	14.67 ± 0.47	121.75 ± 6.73	46.00 ± 0.82	178.28 ± 10.36	80.00 ± 4.89	93.00 ± 5.71	1.75 ± 0.07	0.56 ± 0.02	1.76 ± 0.03	53.74 ± 3.05
SR-157	85.66 ± 2.35	47.26 ± 0.97	6.33 ± 0.47	26.00 ± 1.417	3.267 ± 0.12	2.384 ± 0.13	12.00 ± 0.82	108.55 ± 3.06	38.66 ± 0.47	150.00 ± 4.08	132.33 ± 2.05	23.00 ± 1.63	1.68 ± 0.04	0.46 ± 0.03	1.48 ± 0.02	15.00 ± 0.91
t-value(*d.f*. = 4) n = 3	8.12**	4.26*	4.24*	`0.29NS	2.79*	0.39 NS	4.00*	2.52 NS	11.00***	3.58*	13.93***	16.65***	1.15NS	3.98*	10.93***	17.21***

The values are means of three replicates (n = 3) and ± values indicate standard deviations.

### Measurement of dry weight, soluble sugars and starch

**Figure 1:**
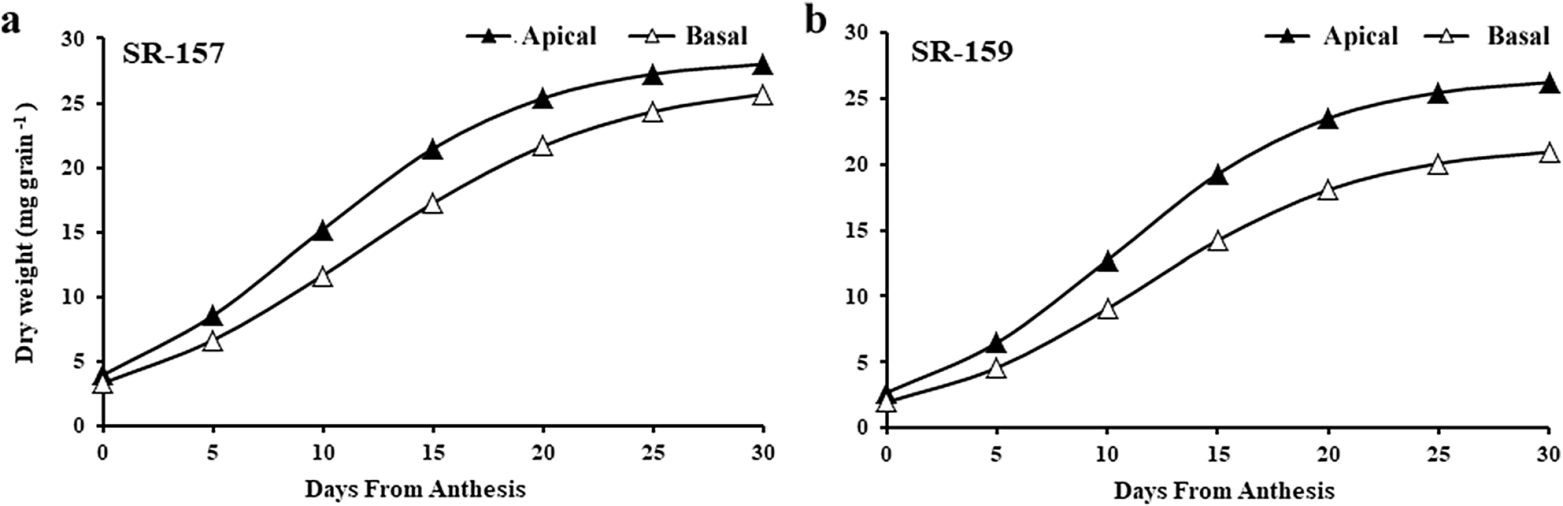
Dry mass accumulation of apical (close symbols) and basal (open symbols) spikelets during post-anthesis period from 0 to 30 days at 5 days interval in SR-157, (**a**) and SR-159, (**b**) The graph has been plotted in terms of logistic growth curve equation, ${\text{W}}\left( {\text{t}} \right) \, = {\text{ Wo M }}/ \, \left[ {{\text{Wo }} + \, \left( {{\text{M }} - {\text{ Wo}}} \right)^{{{-}{\text{kt}}}} } \right]$^48^, where W (t), grain weight at a particular time ‘t’; Wo, initial grain weight; M, maximum grain weight; k, growth rate constant calculated from Microsoft Excel solver, 2007; t, time.

**Figure 2:**
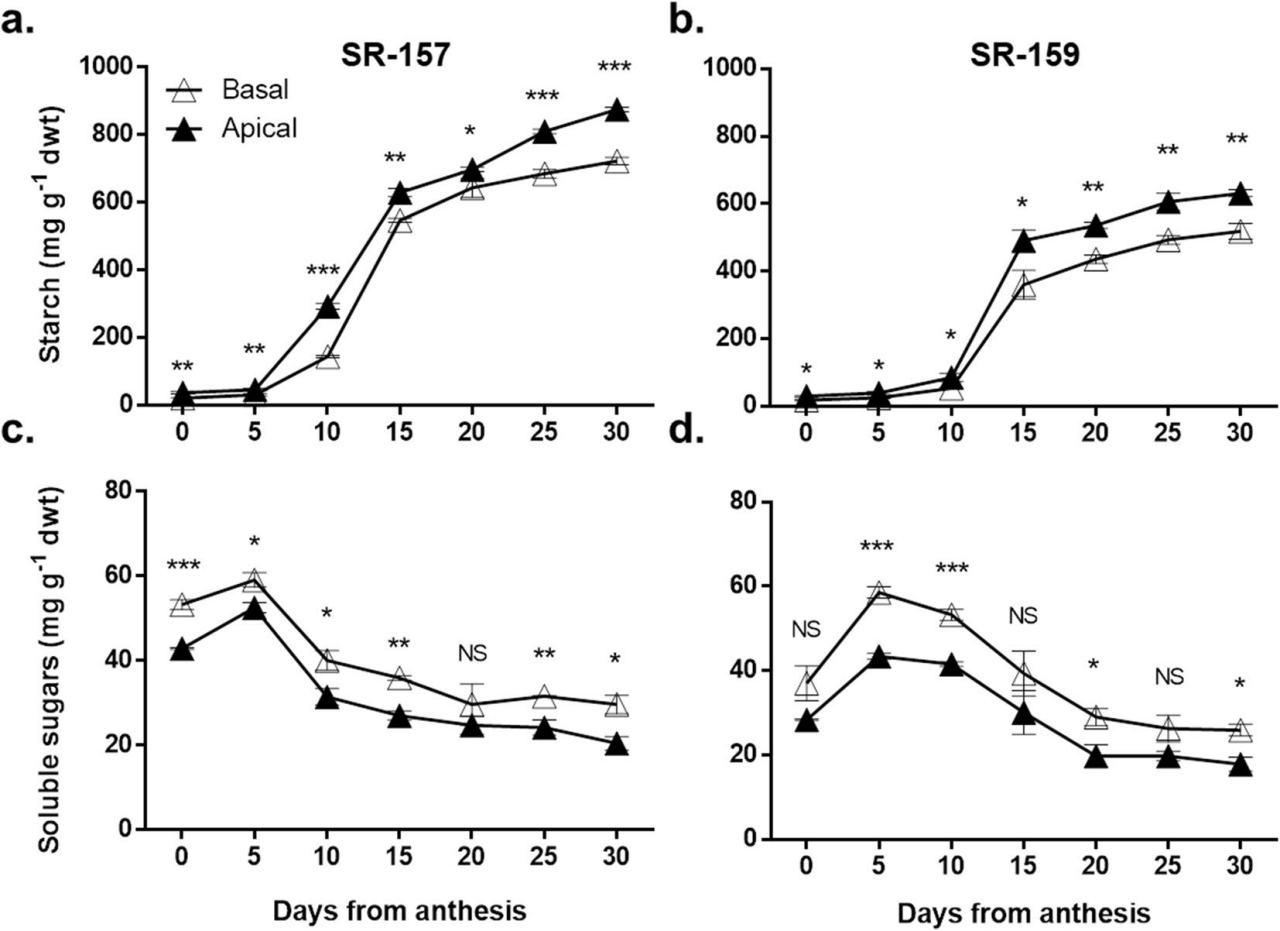
Starch (upper panel: **a**,**b**) and soluble carbohydrate (lower panel: **c**,**d**) concentration of apical (close triangle) and basal (open triangle) of spikelets during post-anthesis period from 0 to 30 days at 5 day intervals in SR-157 (left panel: **a**,**c**) and SR-159 (right panel: **b**,**d**). Individual data represents the mean of 3 replicates (n = 3) and vertical bars represent ± SD values.

The apical and basal spikelet samples of the panicle from the main shoot of the plants were collected at 5 day intervals from day of anthesis (0 day) up to 30 days post-anthesis period and dried inside an oven at 80 °C for 1 h and kept as such at 37 °C for 72 h till constant weight. The dry weight was recorded in an electronic balance and the results were presented in the form of a logistic growth curve

$$ {\text{W}}\left( {\text{t}} \right)= {\text{ Wo M }}/ [{\text{Wo }} + \left( {{\text{M }} - {\text{ Wo}}} \right)^{{{-}{\text{kt}}}} ], $$

where W (t), grain weight at a particular time ‘t’; Wo, initial grain weight; M, maximum grain weight; k, growth rate constant calculated from Microsoft Excel solver, 2007; t, time. The ‘k’ values in apical and basal spikelets of SR-157 are 0.196397 and 0.170065 whereas the values in apical and basal spikelets of SR-159 are 0.209456 and 0.197954, respectively (Fig. 1). The dried samples were subsequently converted into powder form and boiled in aqueous methanol for 10 min and the extract was collected in a volumetric flask. The residue was boiled for second time in 50% methanol and both the extracts were pooled together and volume was adjusted to mark with distilled water. Aliquot of the extract was used for estimation of soluble sugars using Phenol–Sulfuric acid reagent measuring absorbance at 490 nm^24^. The residue after methanolic extraction was boiled with 3% HCl for 3 h in a water bath to convert the starch into glucose and the glucose released was measured quantitatively^24^. The results were expressed in terms of starch by multiplying a factor of 0.9 (Fig. 2).

### RNA isolation, library preparation, sequencing and analysis

Total RNA was extracted from nearly 50 mg fresh weight of rice spikelets after using Spectrum Plant Total RNA Kit (Cat# STRN50-1KT, Sigma) following manufacturer’s instruction. For removal of residual DNA, on-column DNase (Cat#79254, Qiagen RNase free DNase) treatment was done and RNA was eluted in nuclease free water (Cat#AM9939, Ambion, USA). The quantification and quality of the RNA was assessed using Nanodrop 2000 (Thermo Scientific, USA), Qubit (Thermo Scientific, USA) and Bioanalyzer 2100 (Agilent, USA).

Small RNA sequencing libraries were prepared using TruSeq Small RNA Sample preparation protocol (Illumina, San Diego, California, USA) at Genotypic Technology Pvt. Ltd., Bangalore, India. One microgram RNA was used as starting material for the assay. Then 3′ adapters were ligated to the specific 3′OH group of micro RNAs followed by ligation of 5′ adapter. Adapter ligated fragments were reverse transcribed with Superscript III reverse transcriptase (Cat# 18080044, Invitrogen, USA), enriched and indexed by 15 cycles of PCR amplification. The libraries were quantified by Qubit dsDNA HS assay (Thermo Fisher Scientific, MA, USA) and its fragment size distribution was analyzed on Agilent 2100 Bioanalyzer (Agilent Technologies, Santa Clara, USA). The libraries were sequenced using Illumina NextSeq 500 sequencer (Illumina, SanDiego, USA) for 75 bp single read chemistry following manufacturer’s procedure.

### miRNA profiling

Profiling of miRNA was done in the RNA extracts obtained from 12 spikelet samples, six each from groups 1 (SR-159) and 2 (SR-157) plants. Spikelets were sampled from apical and basal portions of the panicle separately on three occasions at 5-day intervals from 0 to 10 days after flowering. Each of these samples was a pooled mixture from spikelets of three individual panicles obtained from the three biological replicates. The 12 RNA samples from two different groups were sequenced followed by length filtering, adapter trimming and elimination having no match. After homology search, the resulting pool of miRNA were categorised into either common or unique miRNA based on their presence in the different days after flowering. Sequencing was done in Illumina single end read chemistry. The raw data of length 75 bp was generated on Illumina platform and received in FASTQ format. Srna workbench V3.0_ALPHA was used to trim adapter and performed length filtering in the range of minimum length 16 bp and maximum 40 bp. To obtain final reads, the exclusion criteria were as follows: (1) low quality reads (< q30), (2) reads without 3′ or 5′ adapters, (3) reads without insert, (4) reads < 16 bp and > 40 bp, (5) reads not matching to reference genome, (6) reads matching to other ncRNAs (rRNA, tRNA, snRNA, snoRNA and piRNA) by referring to NCBI nucleotide database and Rfam version-12 RNA family database which represent multiple sequence alignments, consensus secondary structures and covariance models^25^. Maximum numbers of reads were obtained with sequences of 24 bp (Supplementary Fig. S1). Sequences ≥ 16 bp and ≤ 40 bp length were considered for further analysis. Reads were made unique and hence read count profile was generated (Supplementary Table S1).

### Identification of known miRNAs and their analysis

**Figure 3:**
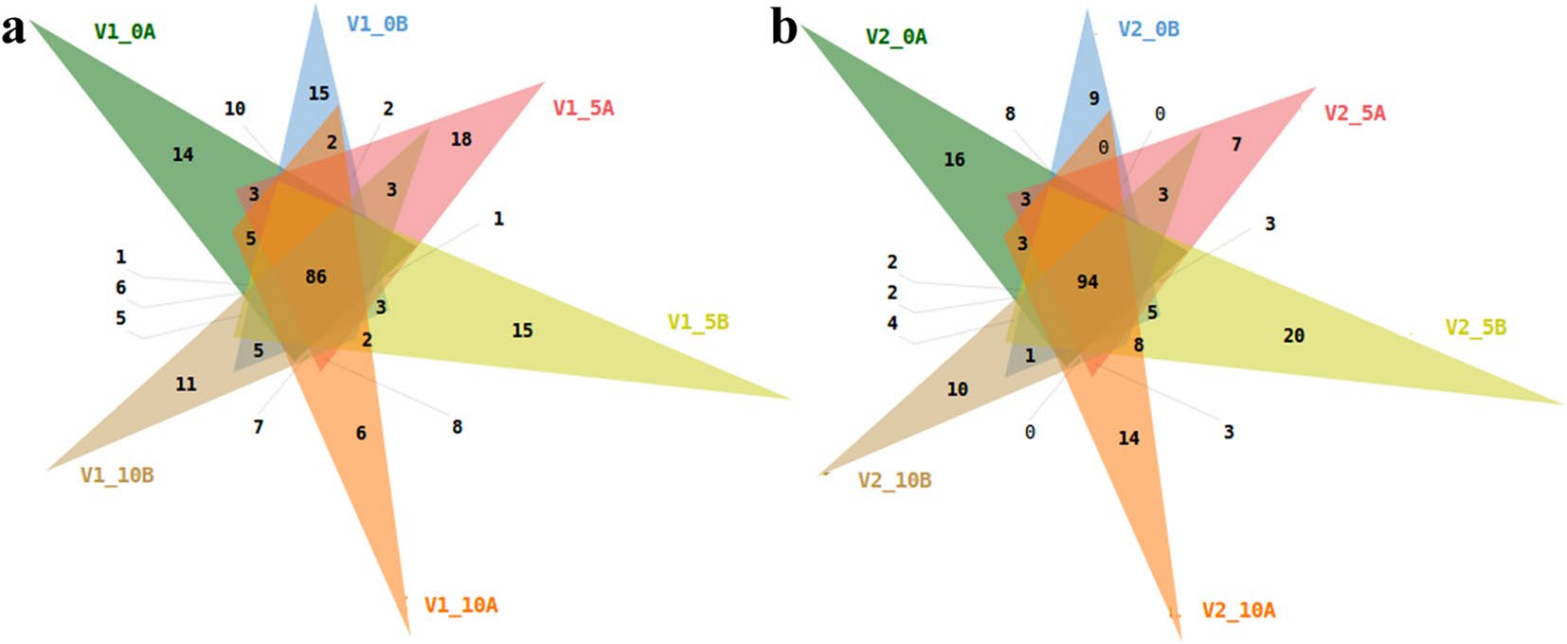
Distribution of common and known miRNAs in a Venn diagram of apical and basal spikelets of V1 (SR-159, **a**) and V2 (SR-157, **b**) during post anthesis (0, 5 and 10 days) in 12 samples based on homology against miRNAs of *Oryza sativa* genome using miRbase-21 database and NCBI-Blast-2.2.30.

The sequences were aligned to *Oryza sativa* sub-subspecies *indica* build genome using Bowtie-1.1.1., which is an efficient short read aligner. The unaligned reads to ncRNAs were used for prediction of known miRNA. Further, homology search of these miRNAs was done against *Oryza sativa indica* mature miRNA sequences retrieved from miRBase-21.3 using NCBI-Blast-2.2.30^26^. The resulted sequences were termed as known miRNAs. All known miRNAs were considered for various analyses and were grouped into 2 categories, i.e. common miRNAs, which were found in both SR-159 and SR-157 cultivars, and unique miRNAs specific to either SR-159 or SR-157. miRNA family analysis revealed that MIR166 family was the most abundant family identified followed by MIR812, MIR444 and others (Supplementary Fig. S2). Total of 86 miRNAs (Fig. 3a) and 94 miRNAs (Fig. 3b) were common among all samples in Group 1 and Group 2, respectively. These reads were aligned to the reference genome using Bowtie1.1.1.

### Differential expression analysis of known miRNA and their fold change

To compare the relative expression patterns and differentially expressed miRNAs, differential gene expression (DGE) analysis was carried out using DESeq8 tool. The variations in the reads were normalized by the library normalization method opted from DESeq library. Mean normalised read counts of the samples in a given condition were used for DGE calculation and heat map generation. Details on differential expression analysis were made according to Anders and Huber^27^. DESeq was used to calculate size factor where the read counts were normalized to transcripts per million (TPM). Normalization was done using following formula:

$$ {\text{Normalized Counts}} = {\text{Raw Count}}/{\text{Size factor}}. $$

**Figure 4:**
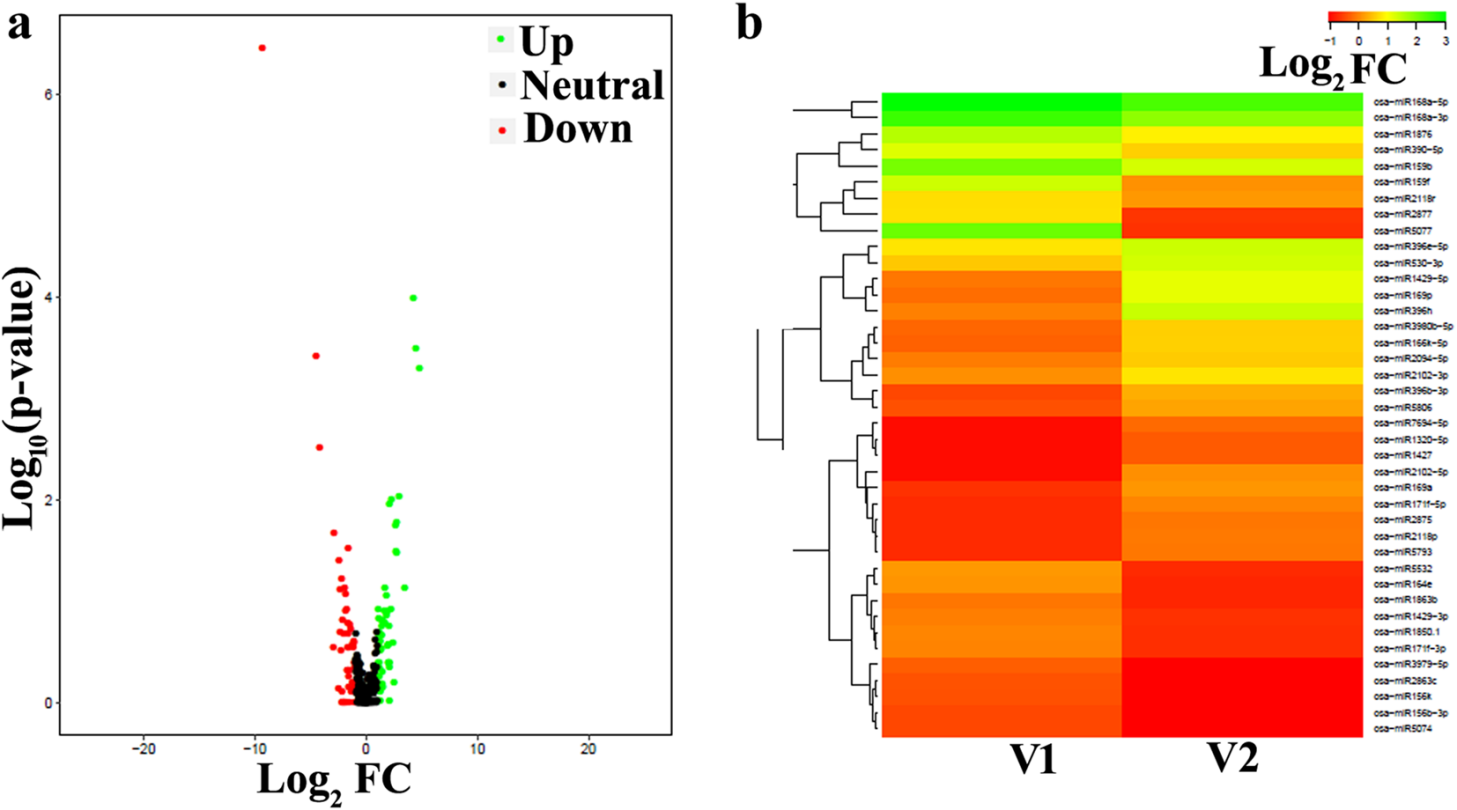
Differential gene expression of miRNAs in 12 samples of V1 (SR-159) and V2 (SR-157) where Log_2_ fold change of 1 was used as cut-off. The miRNAs expression in V1 and V2 are either “UP-regulated (green colour code)” or “DOWN-regulated (red colour code)” in comparison to NEUTRAL-miRNA (black-colour code) represented in scatter plot (**a**) and heat map (**b**).

Size factor was calculated by dividing each column by the geometric means of the rows. The median of these ratios was used as the size factor for this column. For comparison, log_2_ fold of 1 was used as cut-off miRNA, > 1 were considered as up-regulated (UP), miRNAs < − 1 were considered as down-regulated (DOWN) and those between 1 and − 1 were considered as “NEUTRAL”. Heat maps were generated for top 40 miRNA DGEs (top 20 UP and top 20 DOWN according to Log_2_ fold change) among all 12 samples (Fig. 4a). Scale with colour code has been given for SR-159 and SR-157 samples (Fig. 4b).

### Analysis of common miRNAs

For identifying the common miRNAs, DGE related to grain ripening in SR 159 vs. SR 157, each sample of SR-159 (i.e. 0 DFA—Days From Anthesis, apical) was compared with its respective SR-157 (i.e. 0 DFA apical). Likewise, the ratio of fold change (FC) of each miRNA in SR-157 was calculated over that in SR-159 in respective growth stages (Supplementary Table S3). The miRNAs which were reported earlier to be involved in grain filling in rice in other studies^20,22,28,29^ were kept apart, indicated by grey fonts. From the newly identified (49 miRNAs), the miRNAs whose fold change (FC) values changed from +ve to −ve or vice versa were of primary interest in this study (Supplementary Table S3, in bold black fonts).

### Identification of unique miRNAs and target prediction

Unique miRNAs were present in the spikelets of only one of the genotypes (either in SR-159 or in SR-157) while comparison of respective stages of development and hence could not be used for DGE and FC calculation. The pSRNA Target Server (https://plantgrn.noble.org/psRNATarget) and the miRbase (www.mirbase.org) with their default parameters were used to predict the miRNA’s potential targets. The miRNA’s targets that were involved either in ethylene (Supplementary Table S4) or auxin (Supplementary Table S5) signalling responses were short listed. From these lists, miRNA with high read count values were of primary interest for validation.

miRNAs with copy number ≥ 5 were considered for target prediction for known miRNAs. These miRNA sequences were used as input along with reference cDNA and for identification of the potential targets of the known miRNA during the grain filling. *Oryza sativa* MSU Rice Genome Annotation release 6.1 was used for reference.

### Gene ontology analysis

Gene Ontology **(**GO) analysis tool (http://bioinfo.cau.edu.cn/agriGO/analysis.php) was used to emphasize the target gene function of the differentially expressed miRNAs of SR-159 and SR-157 cultivars^30^. Further, the pathways associated with respective miRNA targets and their functional information was obtained from KEGG pathway database (http://www.genome.jp/kegg/). miRNA-target pairs along with GO and KEGG pathway annotation were searched and the input received was used to identify targeted genes of the differentially expressed miRNAs between apical and basal spikelets and also between SR-159 and SR-157 cultivars.

### Quantitative real-time PCR (qRT-PCR) and stem-loop qRT-PCR to validate the identified miRNAs

One microgram of purified RNA was used as starting material for qRT-PCR assay. cDNA synthesis was performed according to Agilent High Specificity 1st strand cDNA synthesis protocol. The assay was performed in Stratagene Mx3005P using Eva Green relative quantification (BioRad) according to manufacturer’s protocol. Data calculation was done using Delta Ct method^31^.

$$ \begin{gathered}   {\text{Fold change of target gene}} = 2^{{ - \Delta \Delta^{\text{CT}}}}  \hfill \\   \Delta {\text{CT }} = {\text{CT }}\left( {{\text{a target gene}}} \right) - {\text{CT }}\left( {{\text{a reference gene}}} \right) \hfill \\  \end{gathered}  $$

Fold change of target gene expression in a target sample relative to a reference sample, normalized to a reference gene.

Ubiquitin primers Os_U51-1a_1 was used against housekeeping miRNA (reference gene) to compare with target sequence (miRNA) for normalization as reported earlier^32,33^. Further, fold change in SR-157 was calculated by comparing the corresponding expressions in SR-159. All primers used in qRT-PCR are listed (Supplementary Table S2).

### Quantitative real-time PCR (qRT-PCR) for Comparative expression analyses of ethylene signal tranducer and auxin homeostasis proteins

Total RNA from apical and basal spikelets of rice genotypes SR-157 and SR-159 at 0 and 5 days post-anthesis was isolated using Trizol (Invitrogen) and was treated with DNase I (Promega) to get rid of genomic DNA contamination which was confirmed by –RT PCR (treated RNA used as template, actin primer set). The genomic DNA-free RNA isolated as above was reverse transcribed using QantiTect Reverse Transcription Kit (Qiagen). The c-DNA prepared was used as template for qRT-PCR analysis by QuantiFast SYBR Green PCR Kit (Thermo Fisher Scientific) on Real Time PCR machine (Applied Biosystem 7500 Fast). Each gene and 18S rRNA as reference control was amplified using gene specific primers designed through Primer Blast. Fold change in gene expression was calculated using 2^−ΔΔCt^^31^. The data presented are mean of three replicates (n = 3). The primer sequence of different ethylene and auxin related genes are given in Supplementary Table S2.

### Ethics approval

The experimental research and field studies on plants, including the collection of plant material, complied with relevant institutional, national, and international guidelines and legislation. The appropriate permissions and/or licences for collection of plant or seed specimens were obtained for the study.

## Results

### Phenotypic differences in grain yield parameters

Plant height and tiller number per plant of the high sterile cultivar SR-159 were larger than that of low sterile cultivar SR-157, but flag leaf area was smaller in the former compared to the latter (Table 1). Both cultivars had similar panicle length and average grain weight, but differed considerably in panicle weight and number of primary and secondary branches per panicle and grain number. Panicle weight was smaller in SR-159 although it had more numbers of primary and secondary branches and grains per panicle. Possession of higher number of grains and branches increased their compactness (density) in the panicle of SR-159. Although panicle spikelet number was high in SR-159, most of them were barren, which decreased the margin of contribution to panicle grain weight more than that of SR-157 cultivar.

The superior spikelets of the apical primary branches had greater dry mass compared to the inferior spikelets of the basal primary branches of panicle in both SR-159 and SR-157 cultivars, but the magnitude of difference in the former was greater than that of the latter (Fig. 1). At anthesis, dry mass of the inferior spikelet was lower than that of superior spikelet. With passage of time, the rate of accumulation of dry mass in the two spikelet types of both cultivars increased progressively and stabilized after day-15 post anthesis showing a sigmoid pattern of growth. The rate of growth was faster in apical than in the basal spikelet. The difference in growth rates of apical verses basal spikelets was greater for high sterile SR-159 than that of low sterile SR-157 (Fig. 1). Although figures not presented, curvilinear growth sports embodying *ln* (natural logarithm) values of grain dry weight (Y-axis) against time scale, i.e. days from anthesis (X-axis) indicated statistical significance of difference between the two spikelet types and cultivars. The equations were encapsulated as follows: SR-159-apical spikelet y = − 0.0031x^2^ + 0.1553x + 1.4101, R^2^ = 0.993, basal spikelet y = − 0.0025x^2^ + 0.1451x + 1.2254, R^2^ = 0.998, SR-157-apical spikelet y = − 0.0036x^2^ + 1.2254 + 0.9956, R^2^ = 0.9948, basal spikelet y = − 0.0034x^2^ + 0.1809x + 0.6915, R^2^ = 0.9962.

### Carbohydrate concentration of spikelets

Similar to dry mass, starch concentration of the developing spikelets increased temporally during the post anthesis period in both the cultivars (Fig. 2a,b). The concentration was higher in apical compared to the basal spikelet. The gradient in concentration between the spikelets was wider in SR-159 than in SR-157. During the post anthesis period, spikelets of SR-159 amassed less starch than that of the cultivar SR-157. Unlike starch, soluble carbohydrate concentration of the developing spikelets decreased with passage of time, after showing an initial increase of level in the first 5 days after anthesis in both the cultivars. The concentration was higher in basal spikelets compared to apical on different stages of development. Between the cultivars, gradient in soluble carbohydrate concentration of apical verses basal spikelet was wider in SR-159 than in SR-157 (Fig. 2c,d).

### Known miRNA analysis

Total unique reads were in the length of 16–40 bp after adaptor trimming, where the percentage of reads aligned to reference genome using Bowtie-1.1.1 exceeded 80 on most of the sampling occasions (Supplementary Table S1). Analyses of clustering reads obtained through homology search done against mature miRNA sequences retrieved from miRbase data base using NCBI-Blast identified 198 known unique miRNA on average belonging to 66 families in the apical and basal spikelets of SR-159 and SR-157 on each sampling occasion in the first 10 days after anthesis (Supplementary Table S1). Between the apical and basal spikelets, the miRNA number was almost similar on days 0 and 10 post anthesis for SR-157, but it differed on day 5, where the number was higher in basal compared to apical spikelet. In SR-159, the number was similar on day 0 post-anthesis between apical and basal spikelets, but it differed on days 5 and 10 post-anthesis. On day 5, miRNA number was higher in apical, and it reversed on day 10. The miRNA family analyses further revealed high abundance of MIR 166 and MIR812 families. It was followed by MIR444 and MIR1846 in a sequence (Supplementary Fig. S2). The number of known miRNAs common to apical and basal spikelets on 0, 5 and 10 days after anthesis were 86 (Fig. 3a) and 94 (Fig. 3b) in SR-159 and SR-157 cultivars, respectively.

### Global analysis of common miRNAs

**Figure 5:**
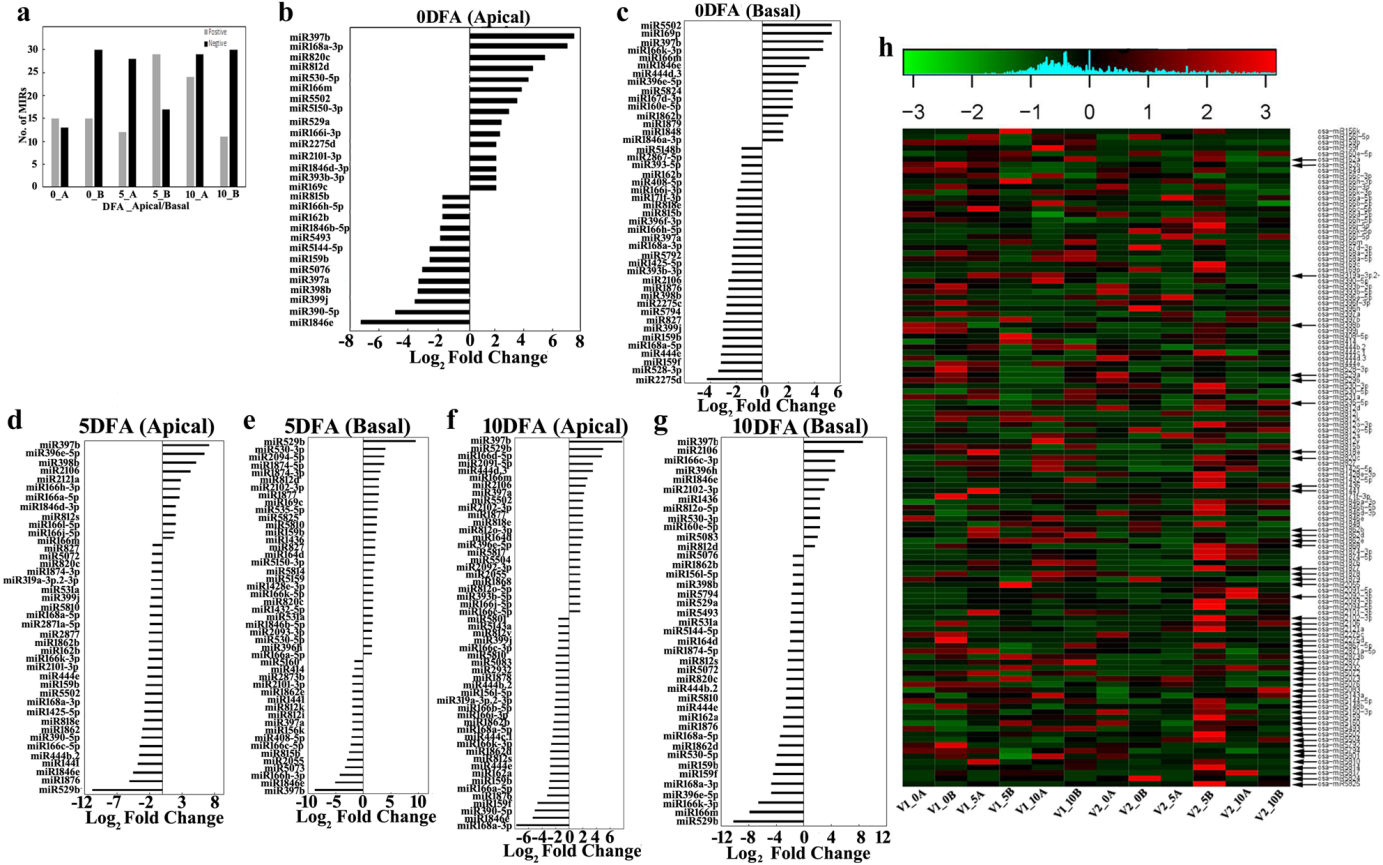
Differential gene expression (DGE) of miRNAs of apical and basal spikelets in V2 (SR-157) over V1 (SR-159) during post-anthesis (0, 5 and 10 days) using DESeq8 tool (**a**). DGE of miRNAs of V2 (SR-157) over V1 (SR-159) based on read length showing UP-regulation or DOWN-regulation after anthesis on 0 day in apical spikelets (**b**), 0 day in basal spikelets (**c**), 5 days in apical spikelets (**d**), 5 days in basal spikelets (**e**), 10 days in apical spikelets (**f**), 10 days in basal spikelets (**g**). Heat map showing DGE analysis of selected miRNAs in apical and basal spikelets of V1 (SR-159) and V2 (SR-157) on days 0, 5 and 10 after anthesis. Here, Log_2_ fold change of 1 was used as cut off, miRNAs > 1 were considered “UP- (red colour code)” regulated, miRNAs < -1 considered “DOWN- (green colour code)” regulated and those between 1 and  − 1 were considered as “NEUTRAL (black colour code)” (**h**).

The comparative study of number of positively and negatively regulated miRNAs of apical and basal spikelets of cultivar SR-157 verses SR-159 exhibited different dynamics with passage of time (Fig. 5a). At anthesis stage the number of positively- and negatively-regulated miRNAs expressing differentially in apical spikelet of SR-157 over SR-159 was almost identical (Fig. 5b). But the situation was not identical for the basal spikelet (Fig. 5c), where the number of negatively expressed miRNA was two times higher. The expression dynamics changed on day 5 post-anthesis. On this occasion, the number of negatively regulated miRNA increased in apical spikelet (Fig. 5d) but not so much in basal spikelets (Fig. 5e). For apical spikelet, the number of miRNA regulated positively or negatively in SR-157 compared with SR-159 reached highest level on day 10 post-anthesis (Fig. 5f,g). On the same occasion, the number of negatively regulated miRNAs differing between the two cultivars was also high in basal spikelet, but in comparison, the number declined considerably for the positively regulated miRNAs. DGE analysis of selected miRNAs in the apical and basal spikelets of SR-159 and SR-157 during 0, 5 and 10 days after anthesis was performed. Heat map generated from this analysis indicated UP-, DOWN- and NEUTRALLY- regulated miRNAs in different developmental stages of grain filling (Fig. 5h).

### Global analysis of unique miRNA

**Figure 6:**
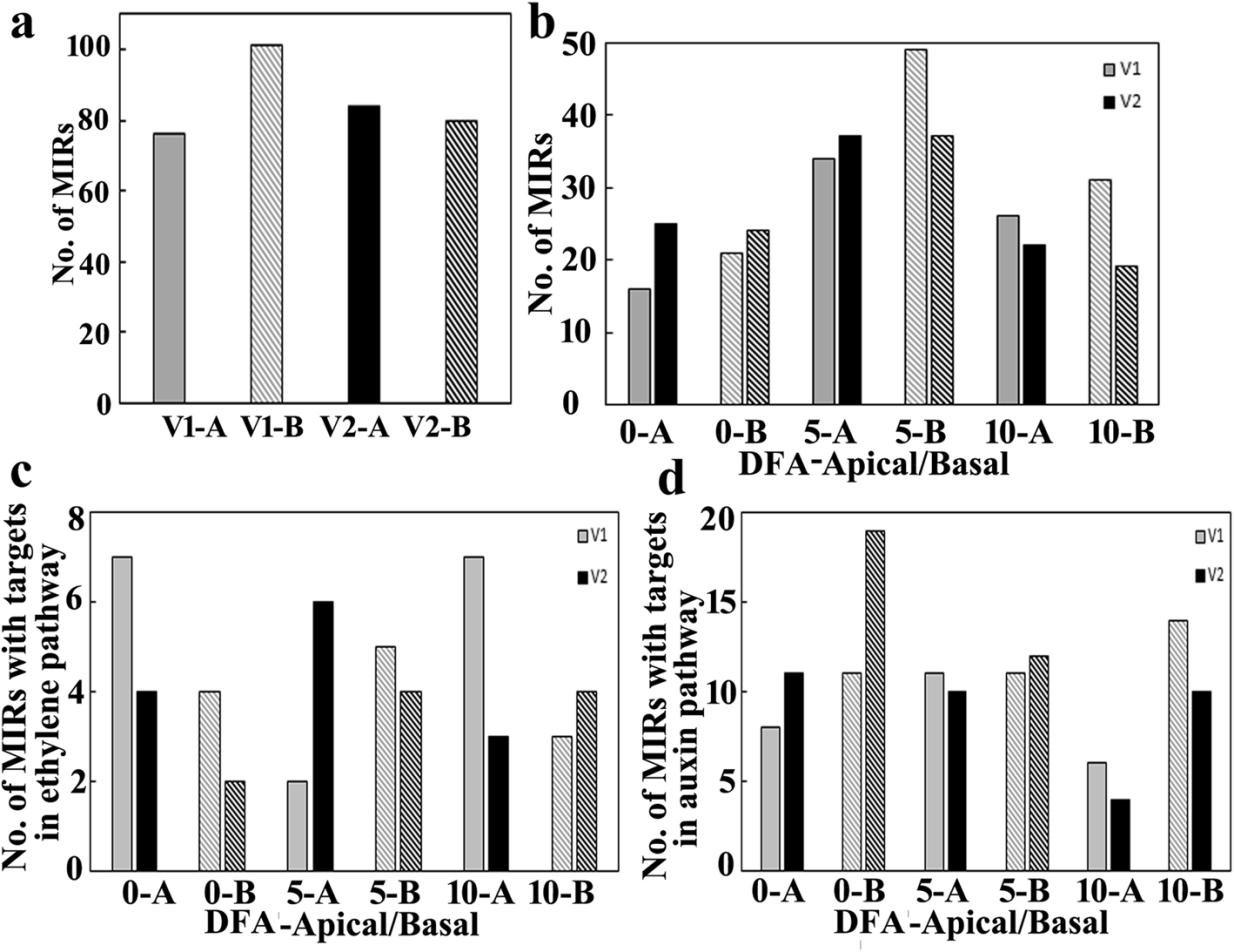
The number of unique miRNAs present in the apical and basal spikelets of either in V1 (SR-159) or V2 (SR-157) during post-anthesis (0, 5 and 10 days) taken together (**a**). The unique miRNA present in the apical and basal spikelets of V1 and V2 (**b**), miRNAs having target genes in the ethylene pathways in the apical and basal spikelets (**c**) and miRNAs having target genes in the auxin pathways in the apical and basal spikelets (**d**) arranged separately during post-anthesis (0, 5 and 10 days).

Differential gene expression analysis for miRNAs in 12 samples of Group1 (SR-159) and Group2 (SR-157) were performed where Log_2_ fold of 1 was used as cut-off. In all these conditions, miRNAs expression were either “UP-regulated” or “DOWN-regulated” in comparison to NEUTRAL-miRNA as represented in scatter plots (Fig. 4a) and heat map (Fig. 4b). After removing number of targets and duplicates, more than 170 miRNA unique to SR-159 genotype were identified during different stages of grain development of the apical and basal spikelets (Fig. 6a). In comparison, less numbers of miRNAs unique to the cultivar were identified for the corresponding spikelets of cultivar SR-157. The targets for the unique miRNAs were searched in the psRNA target server. After removing the number of targets, the number of unique miRNAs found in SR-159 and SR-157 were arranged separately in apical and basal spikelets at different stages of grain filling. The number increased temporally in the first 5 days of anthesis in both apical and basal spikelets of the cultivars and declined thereafter in the following 5 days (Fig. 6b). Between the cultivars the miRNAs showed an overall increase in number in the 10 days period of observation in SR-159, but no corresponding increase of number was recorded for SR-157 during the same period. In SR-159, the number of miRNAs was consistently higher in basal compared to the apical spikelet, but this difference between spikelet types was not exactly discernible in SR-157.

Compared to the unique miRNA searched by psRNA target server, less number of miRNAs with targets in ethylene metabolism pathway was detected (Fig. 6c). In apical spikelet of SR-159, the number of miRNAs declined sharply in the first 5 days of anthesis and recuperated to the same level in the following 5 days. In comparison, the number increased in the basal spikelet in the first 5 days after anthesis and declined later in the following 5 days. At anthesis stage, SR-157 had less number of miRNAs than that of V1 in apical spikelet. The number increased by 50% on day 5 before declining to a level lower than that of the initial position on day 10. For basal spikelet, the number was low at anthesis. It increased by two fold at day 5 and remained unchanged thereafter on day 10. Overall, higher numbers of miRNAs with targets in ethylene pathway were detected in spikelets of SR-159 compared to SR-157 during the period of observation. In SR-159, the number of miRNAs was significantly higher in basal compared to the apical spikelet on day 5 post anthesis and the numerical gradient was not as large between the two spikelet types of SR-157 on this occasion (Fig. 6c).

Compared to ethylene pathway, the number of miRNAs with targets on auxin pathway was at least two times higher (Fig. 6c,d). Between the cultivars, the miRNA number with targets in ethylene pathway was higher in both apical and basal spikelets of SR-159 than in SR-157 on day 0. On day 5, the number was higher in apical spikelet of SR-157 over SR-159, but there was no difference between the cultivars for basal spikelets. The situation changed on day 10 post anthesis, where the number of miRNAs was higher in SR-159 than in SR-157, especially in the apical spikelet.

### Differential gene expression analysis of selected miRNAs

Analysis of common miRNAs between SR-159 and SR-157 was done by studying the fold change of the miRNAs detected in SR-157 in each stage versus their fold change in SR-159 in the respective stages (Supplementary Table S3). A total of 122 common miRNAs including their isoforms were detected to have differential regulation in the 3 different stages of grain development. Out of the total number of 122, expression of 74 miRNAs differed between SR-157 verses SR-159 at anthesis (0 day), 89 on day 5 and 95 on day10 post-anthesis. Highest down- and up-regulation in fold change was found in miRNA529b of the apical and basal spikelets respectively, on day 5 post-anthesis of cultivar SR-157 over SR-159.

A total of 20 miRNAs were moved to a secondary list, which were known in previous reports to be involved in grain filling in rice. Of the new DGEs detected, around 50 miRNAs had the least of single occurrence and 3 miRNAs (namely: miR 159b, miR397b and miR1846e) occurred to a maximum in all the 6 stages. While 46 miRNAs were negatively regulated, 38 miRNAs were positively regulated including all the 3 stages of grain filling among SR-159 vs. SR-157. In 37 miRNAs FC was detected to reverse either from positive to negative or vice versa between SR-159 vs. SR-157 (Supplementary Table S3).

### Target analysis of selected miRNAs

**Figure 7:**
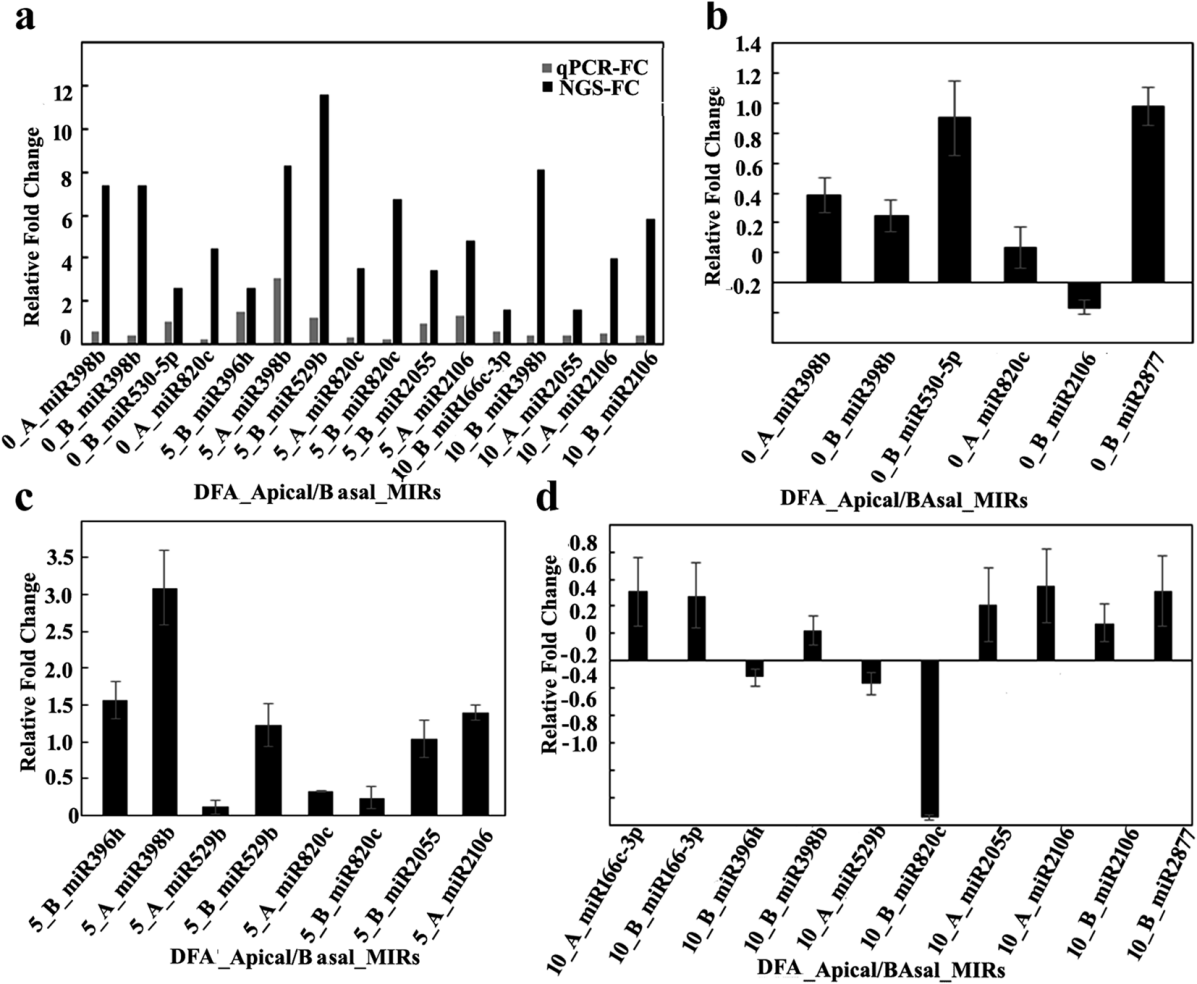
Relative expression of selected miRNAs (miR398b, miR530-5p, miR820c, miR396h, miR529b, miR2055, miR2106, miR166c-3p, miR2877) as per NGS data (Log2 fold-change) (black bar) and relative transcript levels in qRT-PCR (grey bar) (**a**), relative transcript level of 5 miRNAs (miR398b, miR530-5p, miR820c, miR2106, miR2877) on 0 day (**b**), 6 miRNAs (miR396h, miR398b, miR529b, miR820c, miR2055, miR2106) on day 5 (**c**) and 8 miRNAs (miR166c-3p, miR396h, miR398b, miR529b, miR820c, miR2055, miR2106, miR2877) on day 10 (**d**) post-anthesis in the apical and basal spikelets.

Target analysis in the psRNA target server database showed that miRNA398B and miRNA820C have targets respectively, on Os04t0624600-01 (similar to starch synthase DULL1) and Os05t0485800-00 (similar to pollen specific protein NTP303) among the maximum probability prediction (Table 2). On the 5th day, MIR398b and miRNA396h showed highest FC in the apical and basal region respectively (Fig. 7). miR 396 h had multiple targets of Os02t0776900-01, a transcription activator involved in gibberellin induced stem elongation, Os01t0643300-02, an Auxin efflux carrier protein and Os01t0643300-01, a PIN1-like auxin transport protein with maximum probability. On the 10th day, MIR2106 showed highest up-regulation of 1.46 and 1.38 folds in the apical and basal spikelets respectively (Fig. 7c,d). Its target was identified to be Os02t0738900-01, a Dynamin related protein involved in secondary cell wall cellulose biosynthesis (Table 2).

**Table 2:** Selected miRNAs for which qRT-PCR analysis was performed and searched for the target genes in the psRNA target server database.

miRNA	TARGET	P-VALUE	Function
osa-miR398b	Os04t0624600-01	0.0969	Similar to Starch synthase DULL1
osa-miR820c	Os05t0485800-00	0.51939	Similar to pollen-specific protein NTP303
osa-MIR2106	Os02t0738900-01	0.41205	Secondary cell wall cellulose biosynthesis
osa-miR530-5p	Os11t0141000-02	0.11557	Similar to PP2A regulatory subunit-like protein
osa-MIR2877	Os07t0239400-01	0.5	Similar to Ethylene-responsive small GTP-binding protein
osa-miR529b	Os04t0550200-01	0.007149	Pathogenesis-related transcriptional factor and ERF domain containing protein
osa-miR2055	Os09t0439200-01	0.22505	Jasmonate ZIM-domain protein, Jasmonate-induced resistance to bacterial blight, Repressor of jasmonic acid signaling
osa-miR396h	Os02t0776900-01	0.16384	Transcription activator, Gibberellin (GA)-induced stem elongation
osa-miR396h	Os01t0643300-02		Similar to Auxin efflux carrier protein
osa-miR396h	Os01t0643300-01		Similar to PIN1-like auxin transport protein
osa-miR166c-5p	Os02t0759700-03	0.2	Similar to f-box family protein

The most prominent targets of miRNAs, their p-values and important functions are given.

### Validation of NGS expression from comparative expression analyses between SR-159 and SR-157 cultivars in qRT-PCR

Out of the differentially gene expressed (DGE) miRNAs, 9 were selected for validation in qRT-PCR studies mostly based on high read count, higher p values and fold change reversed from positive to negative and *vice-versa* between different stages of grain development (Fig. 7). These miRNAs were namely-miR398b, miR529b, miR530-5p, miR820C, miR2055, miR2106, miR2877, miR396h and miR166c-3p. Fold change values of DGE either positive or negative exhibited similar pattern of expression with that of qRT-PCR analysis. Of the nine selected, 42.3% of the qRT-PCR values had concordance with the fold change values obtained from the NGS analysis. The highest up-regulation of 3.1 fold in the Log_10_ scale was detected in case of MIR398b in apical spikelets of SR-157 on day 5 post anthesis (Fig. 7c). Whereas, highest down-regulation of − 0.96fold in the log 10 scale was detected in case of MIR820c in basal spikelet of SR-157 on 10 day after anthesis (Fig. 7d).

### Comparative expression analyses of ethylene and auxin homeostasis proteins between SR-159 and SR-157 cultivars in qRT-PCR

**Figure 8:**
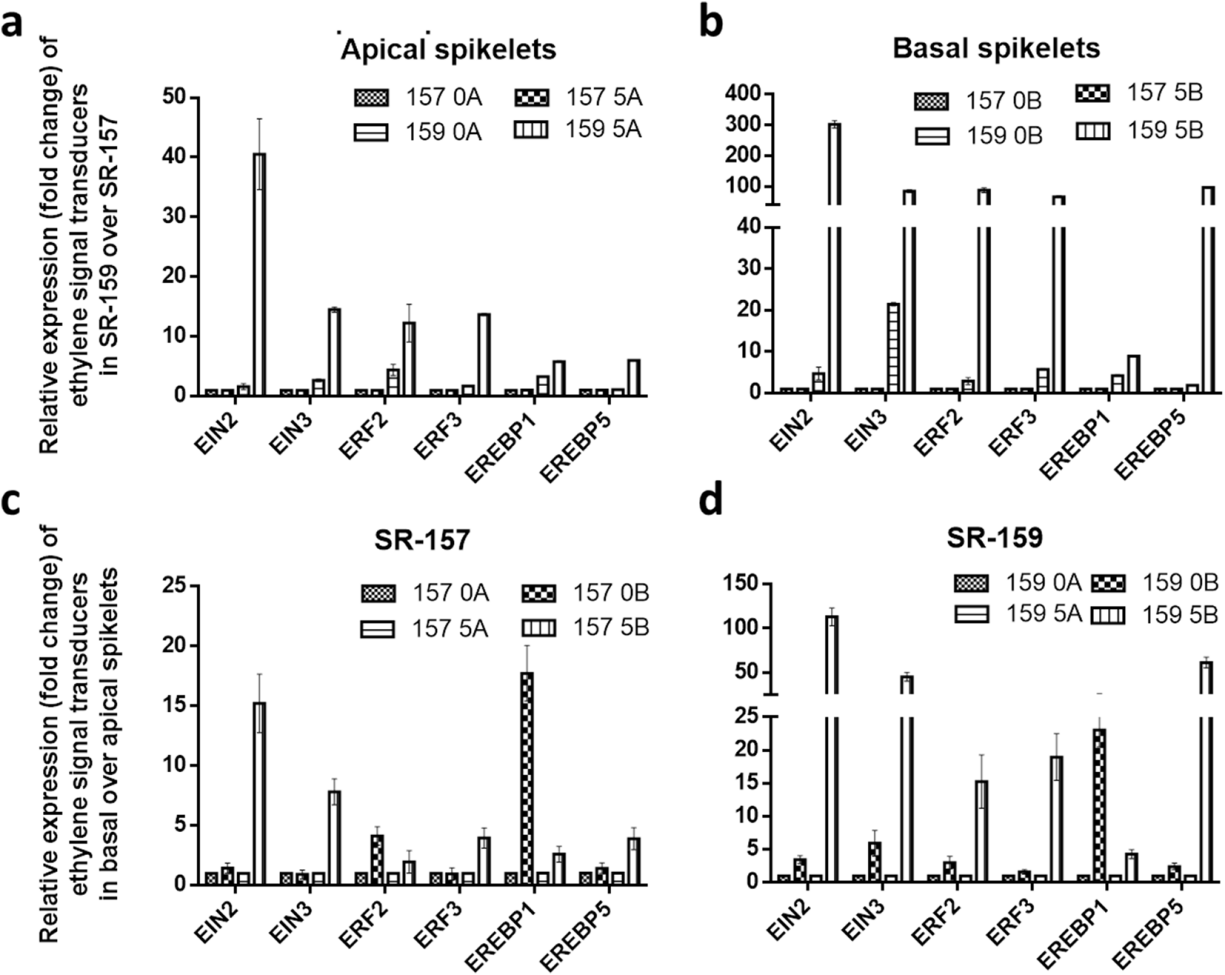
Relative expression (in fold change) of ethylene receptor genes ERS1, ERS2, ETR2, ETR3 and ETR4 in the apical and basal spikelets of the panicle of high sterile (SR-159) over low sterile (SR-157) rice genotypes and in the basal over apical spikelets of SR-157 and SR-159 analysed by Real Time PCR (qRT-PCR). The data are means of three replicates and vertical bars represent ± SD values (n = 3).

**Figure 9:**
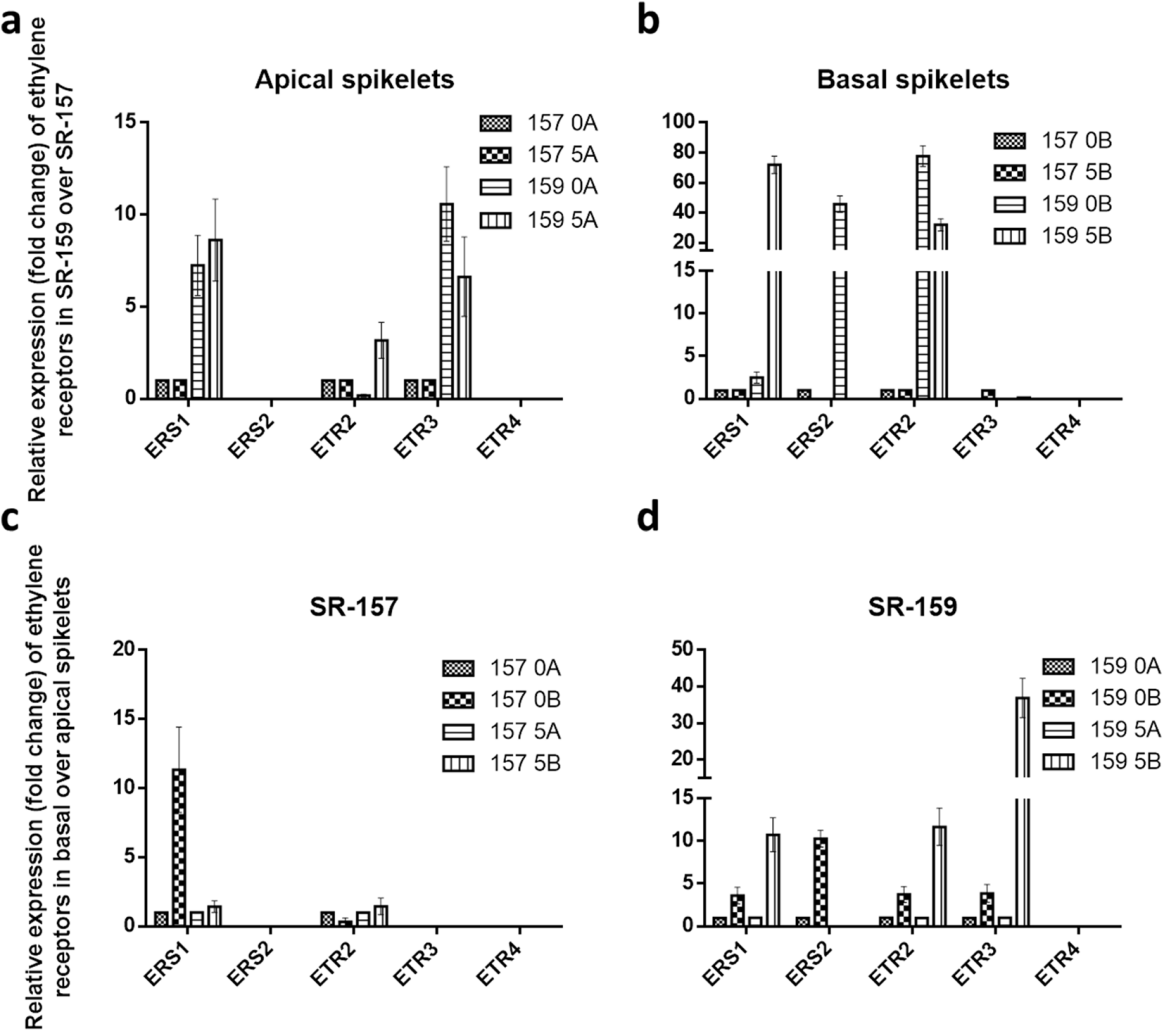
Relative expression (in fold change) of different ethylene signal transducer genes EIN2, EIN3, ERF2, ERF3, EREBP1 and EREBP5 in the apical and basal spikelets of the panicle of high sterile (SR-159) over low sterile (SR-157) rice genotypes and in the basal over apical spikelets SR-157 and SR-159 analysed by Real Time PCR (qRT-PCR). The data are means of three replicates and vertical bars represent ± SD values (n = 3).

**Figure 10:**
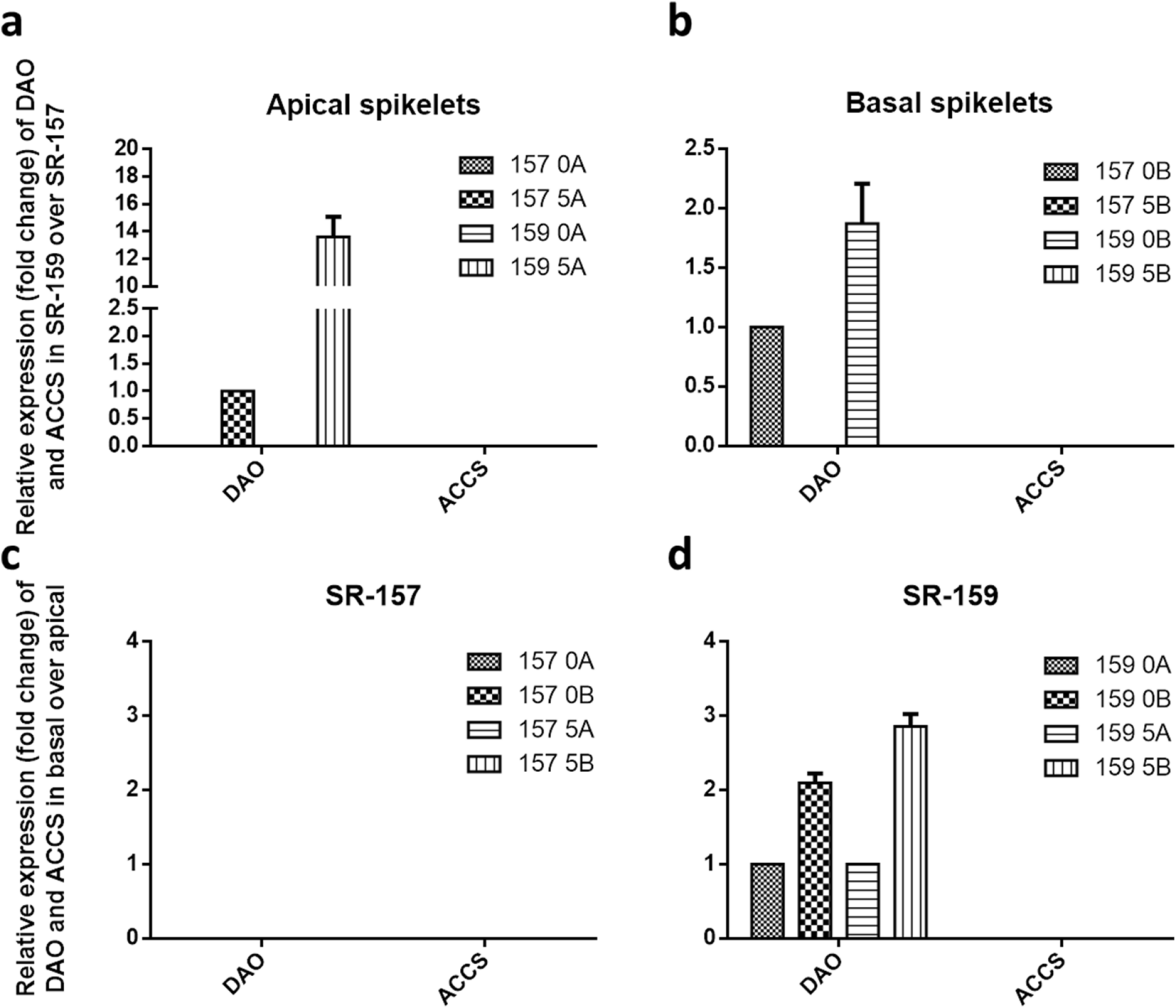
Relative expression (in fold) of auxin oxidising gene DAO (Dioxygenase for Auxin Oxidation) and ethylene synthesizing gene ACCS (ACC synthase) in the apical and basal spikelets of the panicle of high sterile (SR-159) over low sterile (SR-157) rice genotypes and in the basal over apical spikelets SR-157 and SR-159 analysed by Real Time PCR (qRT-PCR). The data are means of three replicates and vertical bars represent ± SD values (n = 3).

In order to confirm regulatory action of MIR on auxin and ethylene dynamics in the early stage grain filling qRT-PCR studies targeting genes for auxin and ethylene synthesis, perception and signal transduction were conducted. Although the expression of auxin pathway genes was not encouraging many of the genes controlling ethylene biology expressed profusely. Genetic expression for ethylene receptors ERS1, ERS2, ETR2 and ETR3 and signal transducers EIN2, EIN3, ERF2, ERF3, EREBP1 and EREBP5 far outstripped in basal corresponding to apical spikelet and cultivar SR-159 in comparison to SR-157 in the first 5 days of grain filling (Figs. 8, 9). The expression increased significantly between 0 and 5 days after anthesis and came to below detection level on day 10 post anthesis. Similar to ethylene receptors and signal carrier proteins, expression DAO (Dioxygenase for Auxin Oxidation) was higher in basal verses apical spikelet and V1 verses V2 cultivar (Fig. 10). According to UniProtKB–Q01IX6 (DAO_ORYSI) BLAST Align Format, DAO is essential for auxin catabolism and maintenance of auxin homeostasis in reproductive organs of *Oryza sativa* subsp. *indica*. The enzyme catalyzes irreversible oxidation of free auxin to the biologically inactive 2-oxoindole-3-acetic acid (OxIAA).

## Discussion

The dynamic expression characteristics of miRNAs between the two extreme types of spikelets, the superior one producing high density grains verses the inferior spikelet failing in grain filling and spikelets of high sterile cultivar against low sterile cultivar have remained elusive so long in the literature. In our previous work, under-expression of genes encoding enzymes/protein factors for starch biosynthesis pathway and endosperm cell division and over-expression of genes controlling synthesis of enzymes, receptors and transducers for up-regulated ethylene synthesis, signal transduction to the detriment of grain filling of inferior spikelet have been identified and explained^12–14,16^. Additionally, it has been shown that high ethylene production slackens grain filling in several rice cultivars^7,8,13^. Obviously, it is evident that expression of genes, which controls hormone homeostasis, endosperm cell division and carbohydrate metabolic pathways are crucial determinants for spikelet specific grain filling in rice panicle at the early stage of grain filling. Precisely, the mechanism of under- and over-expression of the genes, that might have undergone post translational epigenetic modification in the inferior verses superior spikelet and high sterile verses fertile cultivars are not known. In this context, miRNA mediated regulation of grain filling of rice spikelets have been characterized by^20,22,28^. These investigations had specifically targeted the two extreme type of spikelets contrasting for grain filling attributes in a single cultivar *Oryza sativa* L. sub-species japonica, cv. Xinfeng and reached to conclusion that slow filling amounting to poor grain weight of inferior verses superior spikelet could be the final outcome of differences in expressions and functions of miRNAs. In the absence of comparisons between genotypes differing in grain filling phenotype, the conclusion reached above could be unique to the cultivar used.

In the present dispensation scrutiny of miRNA expression involving the two extreme types of spikelets not only in one cultivar but also in cultivars showing sharp contrast in grain filling phenotype were tested and confirmed. In the novelty of our study, two recombinant inbred lines (RIL), namely SR159 and SR157 were used to enable comparison of epigenetic regulation in a narrow genetic window and thereby to preclude interference of genetic factors. Panicle grain yield was not similar between the two RILs primarily because of difference in number of filled grains per panicle (Table 1). But there was no difference in panicle length and average grain weight. The high sterile line SR-159 had more number of spikelets compared to SR-157, but poor grain filling in them (Table1) under-rated filled grain numbers much below its counterpart SR-157. Among the kind of spikelets, inferior spikelets of SR-159 were found most vulnerable for starch synthesis and grain filling. Poor grain sink for starch synthesis owing to sub-optimal activities of regulatory enzymes of the metabolic pathway^10^ resulted in accumulation of soluble carbohydrates in these spikelets. It might have accrued on account of attendant hormonal perceptions, specific to the disadvantaged spikelet.

High throughput miRNA sequencing analysis in the two RILs with contrasting grain filling attributes revealed differential regulation of several miRNAs on a temporal scale during the first 10 days post-anthesis, where dramatic changes in hormone homeostasis is generally noticed in the developing grains. We reported earlier that intrinsic concentration of ethylene is high at anthesis and declines rapidly with passage of time within the first 5 days and comes to ground level thereafter in the residual part of grain development period^7,13,16^. Conversely, intrinsic IAA concentration comes to peak level at 10 days post anthesis and remains stable thereafter till maturity^34^. Jones et al.^35^ opined that endosperm storage capacity (sink capacity) is pre-fixed by cell division, cell expansion and organ proliferation taking place during the early period of grain filling. In rice, we have noted that cell division and starch synthesis of endosperm in rice kernel seldom increase beyond day 12 after anthesis^36^ and hence, genetic or epigenetic factors controlling transcriptome dynamics of the processes are of high value for manipulation of final grain size in this period. Typical to characteristic, miRNAs belong to the class of small RNA possessing base pair length of 20–24^37^. In our study, maximum read length distribution of small RNAs ranged between 23 and 25 bp, with maximum number of sequence at 24 bp (Supplementary Fig. S1), suggesting their participation in grain development. The base pair length of miRNAs involved in grain filling between days 10 and 35 post anthesis of *japonica* rice cv. Jinfeng2 ranges between 20 and 23 bp^22^. While confirming the base pair length of the participatory miRNAs, our study further appends the first 10 days after anthesis into this period for miRNAs epigenetic regulation. An average of 201 and 196 known miRNAs of SR-159 and SR-157 respectively, were detected in cluster analyses during the three stages of grain development in the first 10 days after anthesis (Supplementary Table S1). Similarly, the number of unique miRNAs was at least 10 more in SR-159 than SR-157 (Fig. 6a). However, the number of common miRNAs expressing during the 10 days period after anthesis in SR-159 and SR-157 were 86 and 94 respectively, implying that more epigenetic regulators participated in grain filling of the latter compared to former (Fig. 3). In comparison, Peng et al.^22^ identified 457 known miRNAs in the *japonica* rice cultivar Xinfeng during the grain filling period ranging from day 10 to 35 after anthesis. The sequencing analysis on day 18 in this cultivar revealed presence of 351 and 312 known miRNAs, respectively in the superior and inferior spikelets^21^ and cluster analysis identified 189 miRNAs expressing differentially between the two contrasting spikelets. Taking into consideration, data generated in the aforesaid reports as well as our own, natural expression of these miRNAs in developing rice grain could be specific to the cultivar and sampling stages. In the RIL cultivars used in our study, a total of 66 miRNA families were identified in the developing spikelets during the first 10 days after anthesis. MIR166 and MIR812 families were most abundant in expression followed by MIR444 and MIR1846 (Supplementary Fig. S2). This observation pointed to active participation of MIR166 and MIR812 in targeting gene expression during grain development of the RIL cultivars used, i.e. similar to the observation of Peng et al.^28^, where various members of these two families express significantly during grain filling. In our work, cultivar difference in expression of unique miRNAs was significant, but the number fluctuated with passage of time. Higher number of unique reads of miRNAs aligned to genome expressed on day 5 than on days 0 or 10 post-anthesis and they accounted for at least 80% of the total number of unique reads (Supplementary Table S1). This percentage of distinct reads coinciding to rice genome is much better than the level of 77.18% match observed by Peng et al.^22^. Yi et al.^38^ studied expression profile of miRNAs of developing grains in *Oryza sativa* L. cv. Nipponbare from 5 to 17 days after fertilization and identified 161 known miRNAs expressing differentially during the developmental period and a high proportion of them were up-regulated between days 5 and 7. The known miRNA statistics in our study, almost matched with this observation, in which the expressed miRNA number was often higher on day 5 than on days 0 or 10 after anthesis (Supplementary Table S1).Thus epigenetic regulation of genome for grain filling was most critical at day 5 post anthesis for the cultivars studied.

### Level of miRNA expression in relation to grain filling

It is stated that both types of small RNAs, i.e. miRNAs and siRNAs can regulate expression of mRNA with partially- or fully-complimentary sequences to them at translational or post translational levels^23,39^. miRNAs select and bind to target mRNAs through base pairing leading to their degradation and consequential down gradation of protein translation^40^. Intuitively, it is construed that difference in carbohydrate metabolism, hormone homeostasis and grain filling between superior and inferior spikelets of rice panicle could be the outcome of the differential patterns of expression of miRNAs involved in the processes. With advanced technology, genome-wide miRNA directed target mRNA cleavage have been developed and identified for rice recently^41–43^. In our study DESeq tool was used for DGE analysis during grain filling of the contrasting spikelets of SR-159 verses SR-157 cultivars. GO annotation conducted by Peng et al.^22^, has identified percent of input genes with targets for differentially expressed miRNAs between superior and inferior spikelets with specific functions like development, reproduction and cellular processes. In our study, the potential miRNA target pairs were identified and used for global analysis of positively and negatively regulated DGE with reference to GO enrichment and KEGG pathway annotations. In this process, we identified 122 known miRNAs expressing differentially between the two varieties on the basis of fold change in expression (Supplementary Table S1), out of which 86 and 94 are common to all three stages of development of SR-159 and SR-157, respectively (Fig. 3). Among the differentially regulated common miRNAs, 49 were newly reported. The heat map generated for fold change in expression of 40 selected miRNAs, exhibited drastic up and down regulation dynamics for some of them in the two cultivars; the overall margin of down regulation, judged on the basis of colour intensity chart, was higher for SR-159 than that of SR-157 (Fig. 4b). Peng et al.^22^ have implicated higher expression level of miRNAs positively with grain filling of superior spikelets of rice panicle. It could be possible that similar situation also contributes to varietal difference in grain filling as well. Further, it was noticed that compared to SR-159, miRNA529b expression was down-regulated in apical and up-regulated in basal spikelet of SR-157on day 5 post-anthesis, the most crucial time for grain development. It is surmised that this miRNA might be involved in grain development because of the coincidence of temporal expression at day 5 post anthesis. The expression level was negative in the apical spikelet because it had capacity to produce a well developed high density grain at maturity in both cultivars. Conversely, the miRNA expression was highly positive for basal spikelet of SR-157 because it produced a grain bolder than that of SR-159.

### Genes targeted by differentially expressed miRNAs

The link between miRNA dynamics and traditional role of plant hormones has been proposed to be much complicated^22^. These workers reported that differentially expressed miRNAs, namely miR156, miR149, miR167, miR397, miR1861 and miR1867 target genes related to hormone homeostasis and starch synthesis in rice during grain development. In our study, on a time scale, maximum number of unique miRNA reads was found on day 5 than on days 0 and 10 post anthesis in both SR-159 and SR-157 cultivars (Supplementary Table S1). The total number of unique miRNAs was higher in SR-159 than SR-157 (Fig. 6a) and most of them expressed within the first 5 days after anthesis (Fig. 6b). Between the cultivars, overall expression of unique miRNAs with targets on ethylene pathway genes was higher in high sterile SR-159 than in low sterile SR-157 (Fig. 6c). In particular, the expression at anthesis (day 0) was higher in the former compared to the latter, when ethylene production culminates in rice spikelets resulting grain morbidity^7,13,16^ irrespective of cultivar difference^44^. In comparison, the situation was just opposite for miRNAs with targets on IAA pathway (Fig. 6d). It implied that ethylene evolution was causative for poor grain development and miRNAs participated in its synthesis. We reported earlier that IAA application facilitates development of apical spikelets while compromising with the basal ones^17^ and such discrimination in grain filling might have occurred in the present situation.

Analysis in psTarget server database identified miRNA398B and miRNA820C having targets on Os04t0624600-01 (similar to starch synthase DULL1) and Os05t0485800-00 (similar to pollen specific protein NTP303). Additionally, high expression of miRNA2877 and miRNA530-5p was noticed in basal spikelet of cultivars on day of anthesis. These miRNAs had Os11t0141000-02 and Os07t0239400-01 (PP2A regulatory subunit-like protein and ethylene-responsive small GTP-binding proteins) as targets with highest probability. Irrespective of cultivar difference, ethylene production climaxes at anthesis^44^ and in all probability, these miRNA participated in the process of ethylene biosynthesis for both high and low sterile cultivars used. Further, our study identified expression of MIR396h on day 5 post anthesis, that had multiple targets, such as, Os02t0776900-01, a transcription activator involved in gibberellin induced stem elongation, Os01t0643300-02, an auxin efflux carrier protein and Os01t0643300-01, a PIN1-like auxin transport protein with maximum probability. In contrast to our study, Peng et al.^22^ reported 5 highly conserved miRNA related to auxin homeostasis and signal transduction, namely miR160, miR164, miR167, miR390 and miR393, of which miR164 and miR167 expression is more abundant during grain filling of superior and inferior spikelets of rice panicle. In particular, miR164e expressed 80 and 40 times higher in superior compared to inferior spikelet of rice panicle on days 10 and 15 post anthesis, respectively. In our work, based on high read count, p values and fluctuation dynamics in fold change, the expression of miR396h was higher in basal spikelet of SR-159 compared to SR-157 on day 5 post-anthesis and it was validated in qRT-PCR (Fig. 7). Fold change values of DGE matched with pattern of expression (relative transcript level) in the qRT-PCR studies (Fig. 7) and consolidated strong linkage of miR396h to functions of auxin efflux carrier protein and PIN1-like auxin transport protein; the functions were suppressed in SR-157 compared to SR-159 in the first 5 days of grain filling. There are instances, where perturbation of miR396 regulation greatly increases grain size and yield by modulating development of spikelets through direct induction of the growth regulating factor genes *OsGRF6*^45^ and *OsGRF4*^46^ corresponding to co-ordinate activation of auxin and brassinosteroid biosynthetic genes, respectively. Suppression of miR393 and mi396 might have up-regulated many auxin synthesis and response genes, such as, *YUCCA*, *GH3* and *ARF* for promotion of grain filling by the hormone, although there is scant information on the signaling system^47,48^. In fact studies on hormonal dynamics of grain filling of contrasting spikelets of rice panicle have shown auxin induced growth promotion of apical spikelets at the cost of marginalization of basal spikelets^17^ and ethylene functions as second messenger to auxin for slackening grain growth of the latter^13^.A high auxin concentration at early post anthesis stage can induce an attractive power leading to increased level of cytokinin for promotion of endosperm cell division and stabilisation of sink strength resulting in greater mobilisation of resources for grain filling^49^. It is also possible that attenuated ethylene action generates greater sink strength for grain filling in the dominant apical spikelets because they possess higher ABA concentration and content ratio of ABA/ethylene^50,51^. Profuse up-regulation of expression of genes encoding receptor proteins like ERS1, ERS2, ETR2 and ETR3 and messenger proteins like EIN2, EIN3, ERE2, ERF3, EREBP1 and EREBP5 have consolidated the stance for retrograde action of ethylene in grain filling of the failing spikelets^13^ (Figs. 8, 9). Although robust expression of *DAO* gene has been reported to be essential for auxin homeostasis and seed initiation of rice^52^, our study conflicted with the ideology. Promotion of biologically inactive OxIAA accumulation resulting in the reaction catalyzed by DAO in the disadvantaged spikelets might have precluded beneficial effects of auxins on grain development (Fig. 10).

## Conclusion

In the present study, the intent was to obtain credible information on master modulation of gene expression by miRNAs and their target genes associated with hormonal dynamics that obfuscate processes governing genotype difference of rice cultivars in quantum of grain filling. In pursuit, two etreme type spikelets of panicle, apical and basal, were assessed for epigenetic regulation of grain filling in high sterile SR-159 and low sterile SR-157 RIL lines with reference to genes regulating ethylene and auxin homeostasis. The inferior basal spikelets of SR-159 were most vulnerable to the detrimental action of the hormones that resulted in poor grain filling and thereby widened gradient of grain weight between apical and basal spikelets of cultivar SR-159 compared to SR-157. The study identified gene targets of MIR2877 and MIR530-5p for ethylene and MIR396h for auxin homeostasis pathways at very early post anthesis stage. The credence given to the hypothesis of ethylene induced grain morbidity at this stage is conceptually more invincible because fold change values of DGE and the pattern of expression (relative transcript level) of miRNAs matched in the qRT-PCR studies. In furtherance, the targets of miRNAs on transcriptome dynamics of protein factors controlling ethylene perception and transduction and auxin homeostasis were evaluated for confirmation in qRT-PCR. This information is valuable for promotion of grain filling of disadvantaged spikelets of rice panicle, where morbidity for sometime immediately after fertilization curtails endosperm cell number and final seed size.

## Supplementary Information

Supplementary Information
