# Supplementary material for: MicroRNAs modulate ethylene induced retrograde signal for rice endosperm starch biosynthesis by default expression of transcriptome
Source: Sci Rep. 2021 Mar 10;11:5573. doi: 10.1038/s41598-021-84663-2 (PMC7946924; doi:10.1038/s41598-021-84663-2)
Supplement: Supplementary file 1 — Supplementary Information [file 41598_2021_84663_MOESM1_ESM.pdf]

MicroRNAs Modulate Ethylene Induced Retrograde Signal for Rice Endosperm Starch Biosynthesis by Default Expression of Transcriptome.

Sonam Panigrahi[1#], Madhusmita Panigrahy[2#], Ekamber Kariali[1#], Sushanta Kumar Dash[3#], Binod Bihari Sahu[4#] Sushil Kumar Sahu[5], Pravat Kumar Mohapatra[1+], Kishore Chandra Sekhar Panigrahi[6,7+]

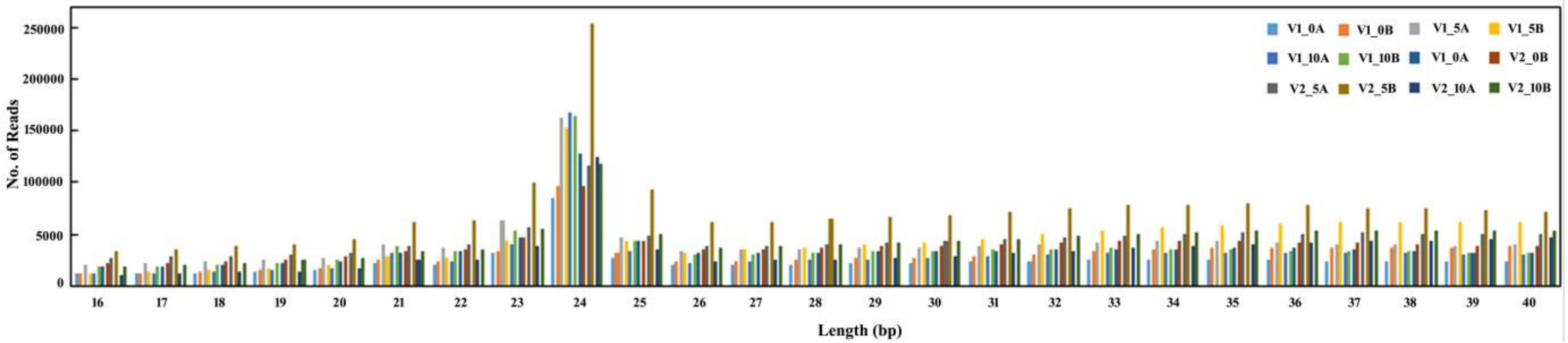

Supplementary Fig 1.

Bar diagram showing read lengths of whole miRNAs in apical and basal spikelets of V1 (SR-159) and V2 (SR-157) during post anthesis (0, 5 and 10 days). All samples name are in figure inlay and distribution pattern in different colour bar diagram.

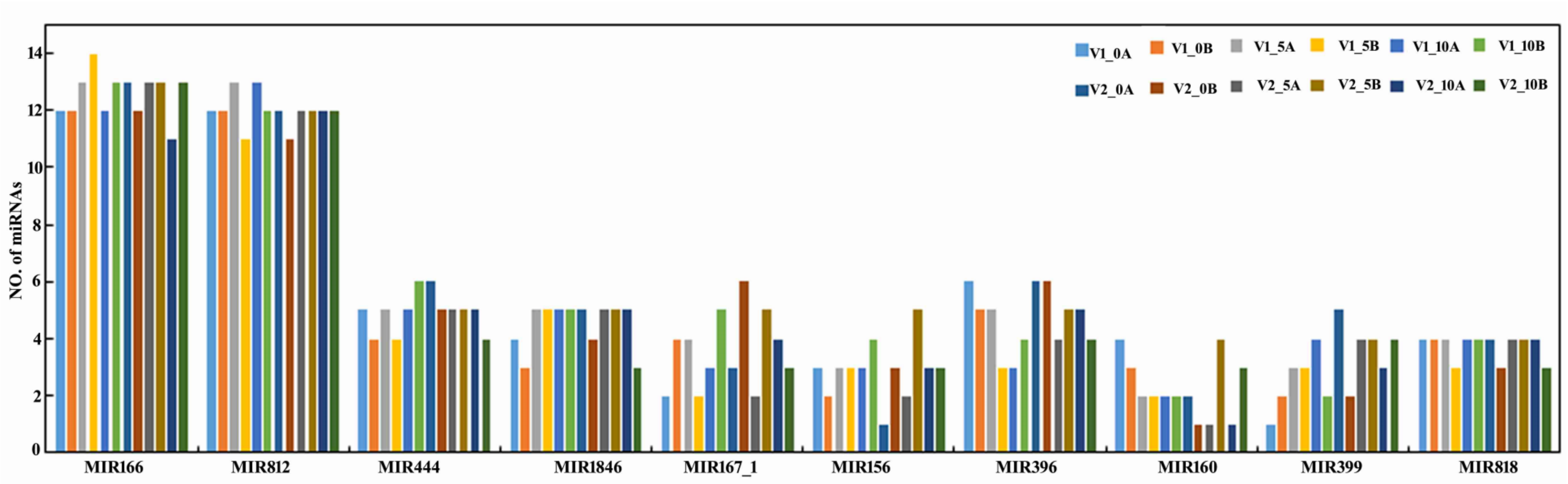

Supplementary Fig 2.

Distribution of 10 best matching families of miRNAs after homology search against *O. sativa* genome. All sequences ≥16 bp and ≤40 bp length were aligned to Rice genome using Bowtie-1.1.1 and miRbase-21.3 database through NCBI-Blast-2.2.30. MIR166 family is most abundant family identified followed by MIR812, MIR444 etc. All samples name are in figure inlay and distribution pattern in different colour bar diagram.

Supplementary Table 1. Read statistics of the miRNAs in the apical and basal spikelets of V1 (SR-159) and V2 (SR-157) samples during post anthesis (0, 5 and 10 days). Trimmed Unique Reads: Total Unique reads in the length of 16-40 after adaptor trimming, Clustered reads: Reads based on 100% coverage and similarity, Reads aligned to miRbase 21.3: Number of reads aligned to mirBase (www.mirbase.org) sequences @ evalue e-3.

	V1_0_A	V1_0_B	V1_5_A	V1_5_B	V1_10_A	V1_10_B	V2_0_A	V2_0_B	V2_5_A	V2_5_B	V2_10_A	V2_10_B
**Total Reads**	12663057	15398985	15214253	15001103	15373790	15533686	11699877	14663099	14151097	27680321	15257092	14821029
**Trimmed Unique Reads**	597863	759393	1055506	1130130	802850	942292	895198	972985	1141823	1840354	844053	1130462
**Reads aligned to genome**	523653	650162	879596	928853	673428	771094	809320	832221	907736	1459010	720568	913939
**Reads aligned to mirBase**	1437	1704	1853	1554	1738	1760	1945	1398	1366	2341	1347	1313
**% reads aligned to genome**	87.59	85.62	83.33	82.19	83.88	81.83	90.41	85.53	79.5	79.28	85.37	80.85
**Known miRNA**	196	199	214	191	191	219	206	194	171	229	190	186
**Unannotated**	4207	6216	8553	6991	6748	8259	5603	6324	8264	14593	5461	7113

Supplementary Table 2. Sequences of primers to amplify selected miRNAs for qRT-PCR analysis (2 a) and primer sequences of genes for different ethylene receptors and signal transducers and enzymes of Auxin (DAO) and Ethylene (ACCS) homeostasis pathways (2 b).

**Sl No**	**miRNA**	**Type**	**Forward primer**
1	Os_U51-1a_1	control miRNA	
2	Os_miR398b_1	Test	5'UGUGUUCUCAGGUCGCCCCUG
3	Os_miR529b_1	Test	5'AGAAGAGAGAGAGUACAGCUU
4	Os_miR820a_1	Test	5'UCGGCCUCGUGGAUGGACCAG
5	Os_miR2055_1	Test	5'UUUCCUUGGGAAGGUGGUUUC
6	Os_miR2106_1	Test	5'CCGAGGUUUUCUGGAUACAUU
7	Os_miR2877_1	Test	5'UUGCAUCCUCUGCACUUUGGGCCU
8	Os_miR530-5p_1	Test	5'UGCAUUUGCACCUGCACCUA
9	Os_miR396d_1	Test	5'UCCACAGGCUUUCUUGAACUG
10	Os_miR166a-3p_1	Test	5'UCGGACCAGGCUUCAUUCCCC

Supplementary Table 3. Relative fold change in the expression pattern of different miRNAs in V2 (SR-157) over V1 (SR-159) samples in 0 day apical, 0 day basal, 5 days apical, 5 days basal, 10 days apical and 10 days basal spikelets. Positive and negative values indicate UP- and DOWN-regulation respectively.

**miRNA**	**Relative Fold Change (FC)**					
	**V2_0_A/V1_0_A**	**V2_0_B/V1_0_B**	**V2_5_A/V1_5_A**	**V2_5_B/V1_5_B**	**V2_10_A/V1_10_A**	**V2_10_B/V1_10_B**
osa-miR156k				-2.000		
osa-miR156l-5p					-2.000	-1.585
osa-miR159b	-2.361	-3.000	-2.656	2.322	-2.923	-4.119
osa-miR159f		-3.170			-4.644	-4.459
osa-miR160e-5p		2.322				2.322
**osa-miR162a**					-2.807	-3.000
**osa-miR162b**	-1.585	-1.585	-2.135			
osa-miR164d				2.170	2.000	-2.000
osa-miR166c-3p					-1.687	4.542
osa-miR166h-3p			2.673	-4.273		
osa-miR166i-3p	1.778	-1.874			-2.170	
osa-miR166k-3p		4.644	-2.187		-2.681	-6.629
osa-miR166a-5p			2.566	1.585	-3.000	
osa-miR166b-5p					-2.170	
**osa-miR166c-5p**			-3.550	-2.322	1.585	

osa-miR166d-5p					4.755	
osa-miR166h-5p	-1.585	-2.000				
osa-miR166j-5p			1.865		1.585	
osa-miR166k-5p				1.807		
osa-miR166l-5p			2.035			
osa-miR166m	3.087	3.585	1.673		2.663	-7.901
osa-miR167d-3p		2.322				
osa-miR168a-3p	5.807	-2.225	-2.812		-7.775	-4.718
osa-miR168a-5p		-3.055	-1.977		-2.383	-3.457
osa-miR169c	1.585			2.644		
osa-miR169p		5.322				
**osa-miR319a-3p.2-3p**			-1.798		-2.048	
osa-miR390-5p	-4.392		-3.250		-5.087	
osa-miR393b-3p	1.585	-2.322				
osa-miR393b-5p		-1.585			1.585	
osa-miR396e-5p		2.722	6.428		1.737	-4.755
osa-miR396f-		-2.000				

3p						
**osa-miR396h**				1.585		4.524
osa-miR397a	-3.000	-2.222		-2.000	2.170	
osa-miR397b	6.172	4.664	7.178	-8.834	7.762	8.542
**osa-miR398b**	-3.036	-2.732	5.151			-1.601
osa-miR399j	-3.248	-3.000	-1.872		-1.585	
osa-miR408-5p		-1.666		-2.135		
osa-miR414				-1.585		
osa-miR444b.2			-3.637		-2.000	-2.524
osa-miR444c.1					-2.459	
osa-miR444d.3		2.807			3.459	
osa-miR444e		-3.170	-2.357		-2.807	-2.585
osa-miR528-3p		-3.369				
**osa-miR529a**	1.874					-1.807
**osa-miR529b**			-10.878	9.533	4.988	-10.237
osa-miR530-3p				4.022		2.322
**osa-miR530-5p**	3.459			1.585		-3.907
osa-miR531a			-1.815	1.682		-1.965
**osa-miR535-5p**				2.585		
osa-miR812d	3.755			2.858		1.585
osa-miR812i				-2.000		

osa-miR812k				-2.000		
osa-miR812o-3p					2.000	
osa-miR812o-5p					1.585	2.322
osa-miR812s			2.035		-2.807	-2.322
osa-miR812v					-1.585	
osa-miR815b	-1.585	-2.000		-2.585		
**osa-miR818e**		-2.000	-2.872		2.000	
**osa-miR820c**	4.459		-1.693	1.755		-2.515
osa-miR827		-3.000	-1.550	2.170		
osa-miR1425-5p		-2.322	-2.872			
osa-miR1428e-3p				1.807		
osa-miR1432-5p				1.700		
**osa-miR1436**				2.170		2.322
**osa-miR1441**			-3.835	-2.000		
osa-miR171f-3p		-2.000				
osa-miR1846a-3p		1.585				
osa-miR1846b-5p	-1.731			1.680		
osa-miR1846d-3p	1.585		2.035			
osa-miR1846e	-6.426	3.322	-4.594	-5.087	-5.358	3.585
osa-miR1848		1.585				

**osa-miR1862b**		2.000	-2.135		-2.322	-1.585
**osa-miR1862d**			-3.135		-2.807	-3.700
**osa-miR1862e**				-2.000		
**osa-miR1868**					1.585	
osa-miR1874-3p			-1.720	3.110		
osa-miR1874-5p				3.781		-2.322
osa-miR1876		-2.585	-5.104		-3.237	-3.026
**osa-miR1877**				2.807	2.000	
**osa-miR1878**					-2.000	
**osa-miR1879**		1.585				
**osa-miR2055**				-2.933	1.585	
osa-miR2091-5p					3.459	
**osa-miR2092-3p**					1.585	
osa-miR2093-3p				1.585		
osa-miR2094-5p				3.807		
osa-miR2101-3p	1.585		-2.357	-2.000		
**osa-miR2102-3p**				2.807	2.000	3.000
**osa-miR2106**		-2.585	4.258		2.415	5.807
**osa-**			2.772			

**miR2121a**						
**osa-miR2275c**		-2.737				
**osa-miR2275d**	1.585	-4.248				
**osa-miR2867-5p**		-1.585				
**osa-miR2871a-5p**			-2.009			
**osa-miR2873b**				-2.000		
**osa-miR2877**			-2.135			
**osa-miR2932**					-2.000	
**osa-miR5072**			-1.653			-2.502
**osa-miR5073**				-3.322		
**osa-miR5076**	-2.807					-1.585
**osa-miR5083**					-2.000	2.000
**osa-miR5143a**					-1.585	
**osa-miR5144-5p**	-2.322					-2.000
**osa-miR5148b**		-1.585				
**osa-miR5150-3p**	2.322					
**osa-miR5148b**		-1.585				
**osa-miR5150-3p**				2.000		
**osa-miR5159**				1.807		

**osa-miR5160**				-1.585		
**osa-miR5493**	-1.739					-1.866
**osa-miR5502**	2.807	5.322	-2.720		2.000	
**osa-miR5504**					1.585	
**osa-miR5792**		-2.322				
**osa-miR5794**		-2.807				-1.807
**osa-miR5801**					-1.585	
**osa-miR5810**			-1.965	2.459	-2.000	-2.585
**osa-miR5814**				1.807		
**osa-miR5817**					1.585	
**osa-miR5824**		2.322				
**osa-miR5825**				2.459		

Supplementary Table 4. Prediction of targets of 'Unique miRNAs' involved in ethylene pathway. The miRNAs unique to V1 (SR-159) or V2 (SR-157) were searched for their targets using miRbase (www.mirbase.org) and pSRNA Target Server (https://plantgrn.noble.org/psRNATarget/email).

miRNA	Targets	Description	P-value
**V1-0A**			
osa-miR1432-5p	Os04t0169100-01	Similar to Ethylene receptor-like protein 1.	0.0789
osa-miR5526	Os02t0820900-02	Similar to Ethylene receptor-like protein 2.	0.5000
osa-miR5526	Os09t0327300-01	Similar to Plastid 5,10-methylene-tetrahydrofolate dehydrogenase (Fragment).	0.5000
osa-miR5526	Os03t0324300-04	Similar to ETHYLENE-INSENSITIVE3-like 1 protein.	0.5000
osa-miR5526	Os03t0324300-02	Similar to ETHYLENE-INSENSITIVE3-like 1 protein.	0.5000
osa-miR5542	Os02t0820900-03	Ethylene receptor, Ethylene response, Floral transition and starch accumulation	0.5000
osa-miR2877	Os07t0239400-01	Similar to Ethylene-responsive small GTP-binding protein.	0.5000
**V1-0B**			
osa-miR160a-5p	Os09t0451400-01	1-aminocyclopropane-1-carboxylate oxidase 1 (EC 1.14.17.4) (ACC oxidase 1) (Ethylene-forming enzyme) (EFE).	0.1027
osa-miR2877	Os07t0239400-01	Similar to Ethylene-responsive small GTP-binding protein.	0.5000
osa-miR167h-3p	Os06t0194000-01	Similar to Ethylene-responsive transcription factor 3 (Ethylene-responsive element binding factor 3 homolog) (EREBP-5) (NtERF5)	0.2500
osa-miR167h-3p	Os06t0194000-02	Similar to Ethylene-responsive transcription factor 3 (Ethylene-responsive element binding factor 3 homolog) (EREBP-5) (NtERF5)	0.2500
**V2-0A**			
osa-miR1428e-3p	Os07t0178100-01	Similar to ETO1-like protein 1 (Ethylene overproducer 1-like protein 1).	0.2500
osa-miR167h-3p	Os06t0194000-01	Similar to Ethylene-responsive transcription factor 3 (Ethylene-responsive element binding factor 3 homolog) (EREBP-5) (NtERF5).	0.1904
osa-miR167h-3p	Os06t0194000-01	Similar to Ethylene-responsive transcription factor 3 (Ethylene-responsive element binding factor 3 homolog) (EREBP-5) (NtERF5).	0.1904
osa-miR160a-5p	Os09t0451400-01	1-aminocyclopropane-1-carboxylate oxidase 1 (EC 1.14.17.4) (ACC oxidase 1) (Ethylene-forming enzyme) (EFE).	0.0481
**V2-0B**			
osa-miR166k-5p	Os07t0155600-01	Nramp ion-transporter family protein, Ethylene signaling pathway	0.1070

osa-miR159a.2	Os02t0764700-01	Similar to Ethylene-responsive transcription factor 4 (Ethylene-responsive element binding factor 4) (Related to APETALA-2 protein 5) (EREBP-4) (AtERF4).	0.2500

V1-5A

osa-miR396a-5p	Os03t0294700-01	Putative E3 ubiquitin ligase, Ethylene-overproduction protein1, Drought and submergence tolerance, Regulation of ethylene synthesis	0.1610
osa-miR5532	Os05t0155200-01	Similar to Ethylene receptor.	0.1619

V1-5B

osa-miR159a.2	Os02t0764700-01	Similar to Ethylene-responsive transcription factor 4 (Ethylene-responsive element binding factor 4) (Related to APETALA-2 protein 5) (EREBP-4) (AtERF4).	0.3673
osa-miR2092-5p	Os05t0155200-02	Similar to Ethylene receptor-like protein (Fragment).	0.8571
osa-miR2092-5p	Os05t0155200-01	Similar to Ethylene receptor.	0.8571
osa-miR2932	Os09t0490200-01	Similar to Ethylene signal transcription factor.	0.3673
osa-miR2877	Os07t0239400-01	Similar to Ethylene-responsive small GTP-binding protein.	0.2222

V2-5A

osa-miR159a.2	Os02t0764700-01	Similar to Ethylene-responsive transcription factor 4 (Ethylene-responsive element binding factor 4) (Related to APETALA-2 protein 5) (EREBP-4) (AtERF4).	0.8452
osa-miR166k-5p	Os07t0155600-01	Nramp ion-transporter family protein, Ethylene signaling pathway	0.1207
osa-miR396c-5p	Os03t0294700-01	Putative E3 ubiquitin ligase, Ethylene-overproduction protein1, Drought and submergence tolerance, Regulation of ethylene synthesis	0.1117
osa-miR2096-5p	Os10t0390800-01	Similar to Ethylene-responsive transcription factor 3 (Ethylene-responsive element binding factor 3) (EREBP-3) (AtERF3).	0.5817
osa-miR2096-5p	Os02t0764700-01	Similar to Ethylene-responsive transcription factor 4 (Ethylene-responsive element binding factor 4) (Related to APETALA-2 protein 5) (EREBP-4) (AtERF4).	0.5817
osa-miR2877	Os07t0239400-01	Similar to Ethylene-responsive small GTP-binding protein.	0.5817

V2-5B

osa-miR1427	Os03t0815800-01	Similar to Ethylene-responsive transcription factor 5 (Ethylene-responsive element binding factor 5) (EREBP-5) (AtERF5).	0.5926

osa-miR2102-5p	Os02t0764700-01	Similar to Ethylene-responsive transcription factor 4 (Ethylene-responsive element binding factor 4) (Related to APETALA-2 protein 5) (EREBP-4) (AtERF4).	0.3951
osa-miR6246	Os09t0327300-01	Similar to Plastid 5,10-methylene-tetrahydrofolate dehydrogenase (Fragment).	0.8889
osa-miR396a-5p	Os03t0294700-01	Putative E3 ubiquitin ligase, Ethylene-overproduction protein1, Drought and submergence tolerance, Regulation of ethylene synthesis	0.2235

V1-10A

osa-miR2877	Os07t0239400-01	Similar to Ethylene-responsive small GTP-binding protein.	0.0947
osa-miR5338	Os02t0202000-00	Similar to ethylene responsive element.	0.5000
osa-miR396a-5p	Os03t0294700-01	Putative E3 ubiquitin ligase, Ethylene-overproduction protein1, Drought and submergence tolerance, Regulation of ethylene synthesis	0.5000
osa-miR414	Os07t0617000-02	Similar to Ethylene response factor 2.	0.5000
osa-miR414	Os07t0617000-01	Similar to Ethylene response factor 2.	0.5000
osa-miR414	Os07t0617000-02	Similar to Ethylene response factor 2.	0.5000
osa-miR414	Os07t0617000-01	Similar to Ethylene response factor 2.	0.5000

V1-10B

osa-miR1432-5p	Os04t0169100-01	Similar to Ethylene receptor-like protein 1.	0.0819
osa-miR2877	Os07t0239400-01	Similar to Ethylene-responsive small GTP-binding protein.	0.0843
osa-miR5532	Os05t0155200-01	Similar to Ethylene receptor.	0.5000

V2-10A

osa-miR1432-5p	Os04t0169100-01	Similar to Ethylene receptor-like protein 1.	0.2500
osa-miR396a-5p	Os03t0294700-01	Putative E3 ubiquitin ligase, Ethylene-overproduction protein1, Drought and submergence tolerance, Regulation of ethylene synthesis	0.1646
osa-miR2932	Os09t0490200-01	Similar to Ethylene signal transcription factor.	0.5000

V2-10B

osa-miR2093-3p	Os03t0324300-05	Similar to ETHYLENE-INSENSITIVE3-like 1 protein.	0.5000
osa-miR2093-3p	Os03t0324300-04	Similar to ETHYLENE-INSENSITIVE3-like 1 protein.	0.5000
osa-miR2093-3p	Os03t0324300-02	Similar to ETHYLENE-INSENSITIVE3-like 1 protein.	0.5000
osa-miR2102-5p	Os02t0764700-01	Similar to Ethylene-responsive transcription factor 4 (Ethylene-responsive element binding factor 4) (Related to APETALA-2 protein 5) (EREBP-4) (AtERF4).	0.5000

Supplementary Table 5. Prediction of targets of Unique miRNAs involved in auxin pathway. The miRNAs unique to V1(SR-159) or V2 (SR-157) were searched for their targets using miRbase (www.mirbase.org) and pSRNA Target Server (https://plantgrn.noble.org/psRNATarget/email).

miRNA	Targets	Target Description	P-value
**V1-0A**			
osa-miR2118q	Os08t0550700-00	Similar to Auxin induced protein.	0.5000
osa-miR2118q	Os04t0662400-01	Auxin responsive SAUR protein family protein.	0.5000
osa-miR5337b	Os04t0442000-01	Similar to Auxin response factor 2 (ARF1-binding protein) (ARF1-BP).	0.5000
osa-miR160f-5p	Os04t0690600-01	Similar to Auxin response factor 13.	0.1527
osa-miR160f-5p	Os06t0685700-01	Similar to Auxin response factor 16.	0.1527
osa-miR160f-5p	Os04t0519700-01	Similar to Auxin response factor 10.	0.1527
osa-miR160f-5p	Os02t0628600-01	Similar to Auxin response factor 8.	0.1527
osa-miR1879	Os01t0768333-00	Auxin responsive SAUR protein family protein.	0.5000
**V1-0B**			
osa-miR160a-5p	Os06t0685700-01	Similar to Auxin response factor 16.	0.1027
osa-miR160a-5p	Os04t0519700-01	Similar to Auxin response factor 10.	0.1027
osa-miR160a-5p	Os02t0628600-01	Similar to Auxin response factor 8.	0.1027
osa-miR160a-5p	Os04t0690600-01	Similar to Auxin response factor 13.	0.1027
osa-miR1863b	Os04t0671900-03	Similar to Auxin response factor 12.	0.2500
osa-miR1863b	Os04t0671900-02	Similar to Auxin response factor 12.	0.2500
osa-miR396f-5p	Os01t0643300-02	Similar to Auxin efflux carrier protein.	0.0556
osa-miR396f-5p	Os01t0643300-01	Similar to PIN1-like auxin transport protein.	0.0556
osa-miR3979-3p	Os09t0491740-01	Auxin efflux carrier domain containing protein.	0.5000
osa-miR444e	Os11t0221000-01	Aux/IAA_ARF_dimerisation domain containing protein.	0.0408
osa-miR5083	Os09t0546400-01	Similar to Auxin induced protein.	0.5000
**V2-0A**			
osa-miR444e	Os11t0221000-01	Aux/IAA_ARF_dimerisation domain containing protein.	0.1904
osa-miR160a-5p	Os06t0685700-01	Similar to Auxin response factor 16.	0.0481
osa-miR160a-5p	Os04t0519700-01	Similar to Auxin response factor 10.	0.0481
osa-miR160a-5p	Os02t0628600-01	Similar to Auxin response factor 8.	0.0481
osa-miR160a-5p	Os04t0690600-01	Similar to Auxin response factor 13.	0.0481
osa-miR2118q	Os08t0550700-00	Similar to Auxin induced protein.	0.5000
osa-miR2118q	Os04t0662400-01	Auxin responsive SAUR protein family protein.	0.5000
osa-miR396f-5p	Os01t0643300-02	Similar to Auxin efflux carrier protein.	0.5000
osa-miR396f-5p	Os01t0643300-01	Similar to PIN1-like auxin transport protein.	0.5000
osa-miR3979-3p	Os09t0491740-01	Auxin efflux carrier domain containing protein.	0.1250
osa-miR5536	Os11t0122800-00	Similar to Auxin Efflux Carrier family protein.	0.5000

**V2-0B**			
osa-miR1861a	Os04t0664400-02	Similar to Auxin response factor 11.	0.5000
osa-miR1861a	Os04t0664400-01	Auxin response factor 1.	0.5000
osa-miR1861a	Os12t0133800-01	Similar to Auxin efflux carrier.	0.5000
osa-miR1861a	Os11t0137000-01	Similar to PIN1-like auxin transport protein.	0.5000
osa-miR1863a	Os01t0919800-01	Similar to Efflux carrier of polar auxin transport.	0.5000
osa-miR2118p	Os08t0550700-00	Similar to Auxin induced protein.	0.2500
osa-miR390-3p	Os09t0442900-01	Similar to Axi 1 (Auxin-independent growth promoter)-like protein.	0.5000
osa-miR396b-3p	Os09t0545400-01	Similar to Auxin induced protein.	0.1406
osa-miR396h	Os01t0643300-02	Similar to Auxin efflux carrier protein.	0.0597
osa-miR396h	Os01t0643300-01	Similar to PIN1-like auxin transport protein.	0.0597
osa-miR5075	Os06t0691400-01	Similar to IAA-amino acid conjugate hydrolase-like protein (Fragment).	0.5000
osa-miR530-3p	Os05t0451900-01	Similar to Axi 1 (Auxin-independent growth promoter)-like protein.	0.0883
osa-miR530-5p	Os06t0702000-01	Auxin responsive SAUR protein family protein.	0.1156
osa-miR530-5p	Os04t0690600-01	Similar to Auxin response factor 13.	0.1156
osa-miR159a.2	Os01t0560000-01	Similar to Auxin amidohydrolase.	0.2500
osa-miR1879	Os01t0768333-00	Auxin responsive SAUR protein family protein.	0.2500
osa-miR5819	Os06t0196700-01	Similar to Auxin response factor 1.	0.5000
osa-miR5819	Os06t0196700-02	Auxin response factor, Transcription factor, Cytokinin-mediated inhibition of phosphate transport, Phosphate signaling, Auxin and phosphate starvation response	0.5000
osa-miR5819	Os06t0691400-01	Similar to IAA-amino acid conjugate hydrolase-like protein (Fragment).	0.5000
**V1-5A**			
osa-miR160e-5p	Os06t0685700-01	Similar to Auxin response factor 16.	0.3850
osa-miR160e-5p	Os04t0519700-01	Similar to Auxin response factor 10.	0.3850
osa-miR160e-5p	Os02t0628600-01	Similar to Auxin response factor 8.	0.3850
osa-miR160e-5p	Os04t0690600-01	Similar to Auxin response factor 13.	0.3850
osa-miR169r-3p	Os02t0628600-01	Similar to Auxin response factor 8.	0.6668
osa-miR169r-3p	Os02t0143400-00	Similar to small auxin up RNA1.	0.6668
osa-miR169r-3p	Os08t0520550-01	Similar to Auxin response factor 21.	0.6668
osa-miR2865	Os02t0164900-01	Similar to Auxin response factor 3.	0.6668
osa-miR2865	Os02t0164900-02	Similar to Auxin response factor 3.	0.6668
osa-miR812q	Os01t0856500-04	Similar to auxin transporter-like protein 1.	0.6531
osa-miR812q	Os01t0856500-03	Similar to auxin transporter-like protein 1.	0.6531
**V1-5B**			
osa-miR156b-3p	Os01t0924400-01	Similar to Auxin-induced basic helix-loop-helix transcription factor.	0.8571
osa-miR159a.2	Os01t0560000-01	Similar to Auxin amidohydrolase.	0.3673
osa-miR1863a	Os01t0919800-01	Similar to Efflux carrier of polar auxin transport.	0.8571

osa-miR1863b	Os01t0221100-01	GH3 auxin-responsive promoter family protein.	0.8571
osa-miR2118n	Os08t0550700-00	Similar to Auxin induced protein.	0.8571
osa-miR2118n	Os04t0662400-01	Auxin responsive SAUR protein family protein.	0.8571
osa-miR2863b	Os06t0603000-01	Heme-oxygenase, Phytochrome chromophore biosynthesis, Nitric oxide- and auxin-induced lateral root formation	0.8571
osa-miR5795	Os11t0186500-00	Similar to GH3 auxin-responsive promoter family protein.	0.8571
osa-miR5819	Os06t0196700-01	Similar to Auxin response factor 1.	0.8571
osa-miR5819	Os06t0196700-02	Auxin response factor, Transcription factor, Cytokinin-mediated inhibition of phosphate transport, Phosphate signaling, Auxin and phosphate starvation response	0.8571
osa-miR3979-3p	Os09t0491740-01	Auxin efflux carrier domain containing protein.	0.6667
**V2-5A**			
osa-miR159a.2	Os01t0560000-01	Similar to Auxin amidohydrolase.	0.8452
osa-miR159a.2	Os11t0247000-01	Similar to Auxin-inducible protein.	0.8452
osa-miR159a.2	Os11t0247000-02	Similar to Auxin-inducible protein.	0.8452
osa-miR1848	Os11t0673200-01	Similar to Auxin-induced beta-glucosidase.	0.8452
osa-miR396b-3p	Os09t0545400-01	Similar to Auxin induced protein.	0.8452
osa-miR396c-5p	Os04t0671900-03	Similar to Auxin response factor 12.	0.1117
osa-miR396c-5p	Os04t0671900-02	Similar to Auxin response factor 12.	0.1117
osa-miR2872	Os02t0557200-01	Similar to Auxin response factor 1.	0.1633
osa-miR5337b	Os04t0442000-01	Similar to Auxin response factor 2 (ARF1-binding protein) (ARF1-BP).	0.5817
osa-miR6248	Os06t0660200-01	Similar to Auxin efflux carrier protein.	0.5817
**V2-5B**			
osa-miR167b	Os04t0671900-03	Similar to Auxin response factor 12.	0.5926
osa-miR167b	Os04t0671900-02	Similar to Auxin response factor 12.	0.5926
osa-miR167b	Os02t0164900-02	Similar to Auxin response factor 3.	0.5926
osa-miR167b	Os12t0613700-02	Similar to Auxin response factor 25.	0.5926
osa-miR167b	Os02t0164900-01	Similar to Auxin response factor 3.	0.5926
osa-miR5144-3p	Os09t0545500-00	Similar to SAUR55 - auxin-responsive SAUR family member.	0.8889
osa-miR529a	Os03t0742800-02	Similar to Auxin-responsive protein IAA14 (Indoleacetic acid-induced protein 14) (SOLITARY-ROOT protein).	0.1756
osa-miR529a	Os03t0742800-01	Similar to Auxin-responsive protein IAA14 (Indoleacetic acid-induced protein 14) (SOLITARY-ROOT protein).	0.1756
osa-miR535-5p	Os08t0118500-00	Similar to Auxin-induced SAUR-like protein.	0.2082
osa-miR535-5p	Os01t0741900-01	Similar to Auxin-responsive protein IAA26 (Indoleacetic acid-induced protein 26) (Phytochrome-associated protein 1).	0.2082
osa-miR5814	Os04t0671900-03	Similar to Auxin response factor 12.	0.2460

osa-miR5814	Os04t0671900-02	Similar to Auxin response factor 12.	0.2460
**V1-10A**			
osa-miR164a	Os04t0459000-01	Multidrug-resistance(MDR)-like ATP Binding Cassette B (ABCB) transporter, P-glyco-protein, Auxin transport, Iron homeostasis	0.2500
osa-miR164e	Os04t0459000-01	Multidrug-resistance(MDR)-like ATP Binding Cassette B (ABCB) transporter, P-glyco-protein, Auxin transport, Iron homeostasis	0.1432
osa-miR2863b	Os06t0603000-01	Heme-oxygenase, Phytochrome chromophore biosynthesis, Nitric oxide- and auxin-induced lateral root formation	0.5000
osa-miR319a-3p	Os02t0628600-01	Similar to Auxin response factor 8.	0.5000
osa-miR5338	Os01t0805500-00	Similar to indole-3-acetate beta-glucosyltransferase.	0.5000
osa-miR5795	Os11t0186500-00	Similar to GH3 auxin-responsive promoter family protein.	0.5000
**V1-10B**			
osa-miR160a-5p	Os06t0685700-01	Similar to Auxin response factor 16.	0.0820
osa-miR160a-5p	Os04t0519700-01	Similar to Auxin response factor 10.	0.0820
osa-miR160a-5p	Os02t0628600-01	Similar to Auxin response factor 8.	0.0820
osa-miR160a-5p	Os04t0690600-01	Similar to Auxin response factor 13.	0.0820
osa-miR166b-5p	Os06t0196700-02	Auxin response factor, Transcription factor, Cytokinin-mediated inhibition of phosphate transport, Phosphate signaling, Auxin and phosphate starvation response	0.5000
osa-miR166b-5p	Os06t0196700-01	Similar to Auxin response factor 1.	0.5000
osa-miR5076	Os08t0554050-01	Similar to auxin-independent growth promoter.	0.2548
osa-miR529a	Os03t0742800-02	Similar to Auxin-responsive protein IAA14 (Indoleacetic acid-induced protein 14) (SOLITARY-ROOT protein).	0.0560
osa-miR529a	Os03t0742800-01	Similar to Auxin-responsive protein IAA14 (Indoleacetic acid-induced protein 14) (SOLITARY-ROOT protein).	0.0560
osa-miR5827	Os06t0335500-02	Similar to Auxin-responsive protein IAA21.	0.2548
osa-miR5827	Os06t0335500-01	AUX/IAA protein family protein.	0.2548
osa-miR810b.1	Os04t0517900-01	Auxin responsive SAUR protein family protein.	0.1166
osa-miR810b.1	Os01t0221100-02	GH3 auxin-responsive promoter family protein.	0.1166
osa-miR810b.1	Os01t0221100-01	GH3 auxin-responsive promoter family protein.	0.1166
**V2-10A**			
osa-miR166b-5p	Os06t0196700-02	Auxin response factor, Transcription factor, Cytokinin-mediated inhibition of phosphate transport, Phosphate signaling, Auxin and phosphate starvation response	0.5000
osa-miR166b-5p	Os06t0196700-01	Similar to Auxin response factor 1.	0.5000
osa-miR1846d-5p	Os06t0671150-00	Auxin responsive SAUR protein family protein.	0.1646

osa-miR171f-5p	Os12t0601300-01	Similar to Auxin-responsive protein (Aux/IAA) (Fragment).	0.5000
**V2-10B**			
osa-miR160e-5p	Os06t0685700-01	Similar to Auxin response factor 16.	0.1245
osa-miR160e-5p	Os04t0519700-01	Similar to Auxin response factor 10.	0.1245
osa-miR160e-5p	Os02t0628600-01	Similar to Auxin response factor 8.	0.1245
osa-miR160e-5p	Os04t0690600-01	Similar to Auxin response factor 13.	0.1245
osa-miR160e-5p	Os04t0459000-01	Multidrug-resistance(MDR)-like ATP Binding Cassette B (ABCB) transporter, P-glyco-protein, Auxin transport, Iron homeostasis	0.1245
osa-miR166a-5p	Os04t0662400-01	Auxin responsive SAUR protein family protein.	0.1245
osa-miR396h	Os01t0643300-02	Similar to Auxin efflux carrier protein.	0.0435
osa-miR396h	Os01t0643300-01	Similar to PIN1-like auxin transport protein.	0.0435
osa-miR5536	Os11t0122800-00	Similar to Auxin Efflux Carrier family protein.	0.5000
osa-miR166h-5p	Os06t0671150-00	Auxin responsive SAUR protein family protein.	0.2500

Website link: Micro-RNA sequencing data has been submitted at NCBI GEO server with SRA submission ID: SUB5947694, bio project no: PRJNA553304 and can be viewed at the following link: https://dataview.ncbi.nlm.nih.gov/object/PRJNA553304?reviewer=2aahokaanulp7us6lq1a63j31p
